# Novel *Klebsiella pneumoniae* and *Pseudomonas aeruginosa* MAPS vaccine combining O polysaccharides and pathogen-specific proteins

**DOI:** 10.1128/mbio.00807-25

**Published:** 2025-06-26

**Authors:** Mohammed N. Amin, James E. Sinclair, Brittany Curtis, Lada Sycheva, Heather Fox, Myeong-Jin Choi, Surekha Shridhar, Jacqueline Kolasny, Joseph Nkeze, Ethel Apolinario, Sang Hyun, Nicolas Hegerle, Shaichi Sen, Jasnehta Permala Booth, Mark Leney, Deborah Molrine, Greg Saia, Marcela Pasetti, Teresa Broering, Francis Michon, Richard Malley, George R. Siber, Sharon M. Tennant, Donna Ambrosino, Raphael Simon, Alan Cross

**Affiliations:** 1 Center for Vaccine Development and Global Health, University of Maryland School of Medicine 12264, Baltimore, Maryland, USA; 2 Affinivax Inc. (a GSK company), Cambridge, Massachusetts, USA; 3 RRD International 376190 , Rockville, Maryland, USA; 4 Nosocomial Vaccines Company, Rockville, Maryland, USA; 5 Siber Biotechnologies LLC, New York, New York, USA; University of Wisconsin-Madison, Madison, Wisconsin, USA

**Keywords:** *Klebsiella pneumoniae*, *Pseudomonas aeruginosa*, MAPS vaccine, O polysaccharide, MrkA, PcrV, flagellin

## Abstract

*Klebsiella pneumoniae* (KP) and *Pseudomonas aeruginosa* (PA) are leading causes of healthcare-associated and community-acquired infections. Despite decades of efforts, there are no licensed vaccines for either pathogen. The surface O polysaccharides (OPS) of *Klebsiella pneumoniae* and *Pseudomonas aeruginosa* are essential virulence factors for these bacteria and are targets of protective antibacterial antibodies. The multiple antigen presenting system (MAPS) vaccine platform was used to create a multivalent *Klebsiella*/*Pseudomonas* vaccine in which the surface OPS of four *Klebsiella* and eight *Pseudomonas* strains were linked to a high MW biotinylated *Klebsiella oxytoca* (KO) K19 capsular polysaccharide backbone and subsequently linked to pathogen-specific fusion proteins, consisting of *Klebsiella* MrkA, the *Pseudomonas* flagellin B D2 subunit, and PcrV. We show here that these MAPS complexes incorporating KP and PA OPS, as well as pathogen-specific proteins, were highly immunogenic in rabbits and mice and induced functionally active antibodies *in vitro* and in murine animal models.

**IMPORTANCE:**

Infections caused by *Klebsiella pneumoniae* (KP) and *Pseudomonas aeruginosa* (PA) are widely recognized to be of “urgent” and “serious” concern, in part because of their widespread antimicrobial resistance. To date, there is no licensed vaccine for either pathogen. We developed a novel multiple antigen presenting system (MAPS) vaccine platform that contains eight O polysaccharides (OPS) of PA and four OPS of KP, which will cover over 80% of clinical infections with these pathogens. In addition, this vaccine includes three pathogen-relevant proteins acting both as carrier proteins to provide T cell help for these polysaccharides as well as to elicit functionally active antibodies that may also protect against KP and PA infection. The use of pathogen-relevant proteins that may contribute to vaccine efficacy in place of the more traditional carrier proteins is a novel concept. The MAPS vaccine platform also generates Th17 and Th1 responses. This KP/PA MAPS vaccine may prevent infection with these pathogens and reduce their transmissibility.

## INTRODUCTION

Nosocomial infections are a prominent threat for both acute care and elective procedures in healthcare settings, and the specter of antibiotic resistance has complicated treatment and prevention by conventional antibiotic regimens. Multistate single-day point-prevalence surveys representing large hospitals across the USA have found that 10 bacterial pathogens account for over 77% of all nosocomial infections (1). Among these, two gram-negative bacteria, *Klebsiella pneumoniae* (KP) and *Pseudomonas aeruginosa* (PA), cause ~17% of all healthcare-associated infections (HAI), including surgical site infections, pneumonias, urinary tract infections, bloodstream infections, and central line infections (1). Importantly, these infections frequently occur in the context of prophylactic antibiotic administration. Widespread antimicrobial resistance (AMR) has been reported among clinical isolates of both PA and KP, driven in part by the transfer of plasmid-based antibiotic resistance genes. Of particular concern is the appearance and rapid rise in the prevalence of KP and PA strains that are resistant to carbapenems and colistin.

Immunoprophylactic approaches for the prevention of KP and PA infections represent a promising countermeasure to the threat of HAIs. The surface polysaccharides of these bacteria have been identified as essential virulence factors and promising protective antigens (2–4). Lipopolysaccharide (LPS) contains lipid A and a conserved core polysaccharide that is itself linked at its non-reducing end to a polymer of O polysaccharide repeats for which differences in the chemical structure are serotype-defining. *Klebsiella* can express both a lipopolysaccharide (O antigen) and a capsular polysaccharide (CPS, K antigen). There are more than 80 identified CPS types, at least 20 of which would be required for 60–80% coverage (5). By comparison, there are only eight recognized KP OPS types, of which four types (O1, O2a, O3, and O5) predominate among clinical isolates worldwide (6). KP OPS are important virulence factors and protective antigens that are the targets of antibacterial antibodies (4, 7, 8). PA can express up to three different exopolysaccharides (alginate, Psl, and Pel), as well as LPS. Antibodies against PA LPS have protected animals in challenge models, and naturally acquired anti-PA OPS antibody levels have been associated with protection against PA infections in humans (9–11). The International Antigenic Typing System (IATS) recognizes 21 different PA OPS chemotypes, but a subset of only eight chemotypes (O1, O2, O3, O4, O5, O6, O10, and O11) would be sufficient to provide coverage for 60–80% of infections (12).

Immunoprophylactic approaches for the prevention of KP and PA infections represent a promising countermeasure to the threat of HAIs, and vaccines against these pathogens are in active development. Previously, a multivalent O polysaccharide/exotoxin A conjugate and outer membrane protein fusion protein vaccines for *Pseudomonas* had advanced through clinical development but are no longer being pursued (13, 14). Flagellin- and PcrV-based experimental PA vaccines have been extensively tested in pre-clinical animal models with encouraging results (15–17). With KP becoming an increasingly important global pathogen, there is considerable effort, with a variety of platforms, toward the development of a vaccine for KP. Given its importance as a KP virulence factor, a 24-valent CPS KP vaccine was studied several decades ago, but there is renewed interest in developing a multivalent KP CPS vaccine (5, 18). This effort is compromised by the number of CPS PS that would be needed to achieve adequate clinical coverage. Since most KP infections are caused by only four KP O serotypes (19), OPS-based KP glycoconjugate and bioconjugate vaccines are being developed, the latter having advanced to clinical testing (4, 20). Outer membrane vesicle vaccines (21) that express highly conserved surface antigens of interest are under active development. The type III fimbriae of KP, MrkA, have shown promise in preclinical studies (22). A more detailed review of KP, PA, and other gram-negative bacterial vaccines is available (23). In addition to vaccines for monoprophylaxis, there has been much work on mAbs and phage cocktails for the treatment of these infections.

Bacterial polysaccharides are generally type I thymus-independent antigens and thus poor immunogens that do not induce immunologic memory, boosting, or affinity maturation. Linkage to carrier proteins has enabled T-cell help, and bacterial conjugate vaccines have been highly immunogenic at all ages. As vaccine constructs, conventional glycoconjugates have several limitations, however. Most licensed conjugate vaccines utilize carrier proteins that are already administered as part of routine immunization schedules, such as tetanus toxoid, or genetically or chemically inactivated derivatives of diphtheria toxin. Conventional conjugation approaches also employ linkage to random reactive groups on both the polysaccharide and the protein carrier that can lead to uncontrolled disruption of putative protective epitopes (24). The multiple antigen presenting system (MAPS) platform overcomes these limitations by non-covalently linking biotinylated polysaccharides with a fusion protein of an avidin family member genetically linked to a pathogen-specific protective antigen (25). The resulting complex is highly stable and offers site-specific linkage that preserves the functional epitopes on the protein. The MAPS approach has been used successfully to enhance the immunogenicity of high-molecular-weight capsular polysaccharides, such as those of *Streptococcus* (26, 27), the Vi capsular polysaccharide of *Salmonella enterica* serovar Typhi, and the OPS of *S. enterica* Paratyphi A (28). KP and PA OPS, by comparison with capsular polysaccharides or even the OPS of *S. enterica* Paratyphi A, are much smaller molecules, a drawback that limits the ability to form high-molecular-weight complexes. To adapt the MAPS system for use with small OPS in a manner that permits the formation of large multimeric complexes, we utilized a novel strategy wherein a pathogen-specific high MW KP capsular polysaccharide was used as the backbone to which both OPS molecules and biotin were chemically linked. Protein:polysaccharide complex formation along this backbone occurs following incubation with rhizavidin fusion proteins. We report herein the synthesis and characterization of these backbone MAPS complexes incorporating KP and PA polysaccharides and proteins, as well as their immunogenicity and induction of functional antibacterial antibodies in animal models.

## RESULTS

### Production and characterization of vaccine polysaccharides and backbones

**Fig 1:**
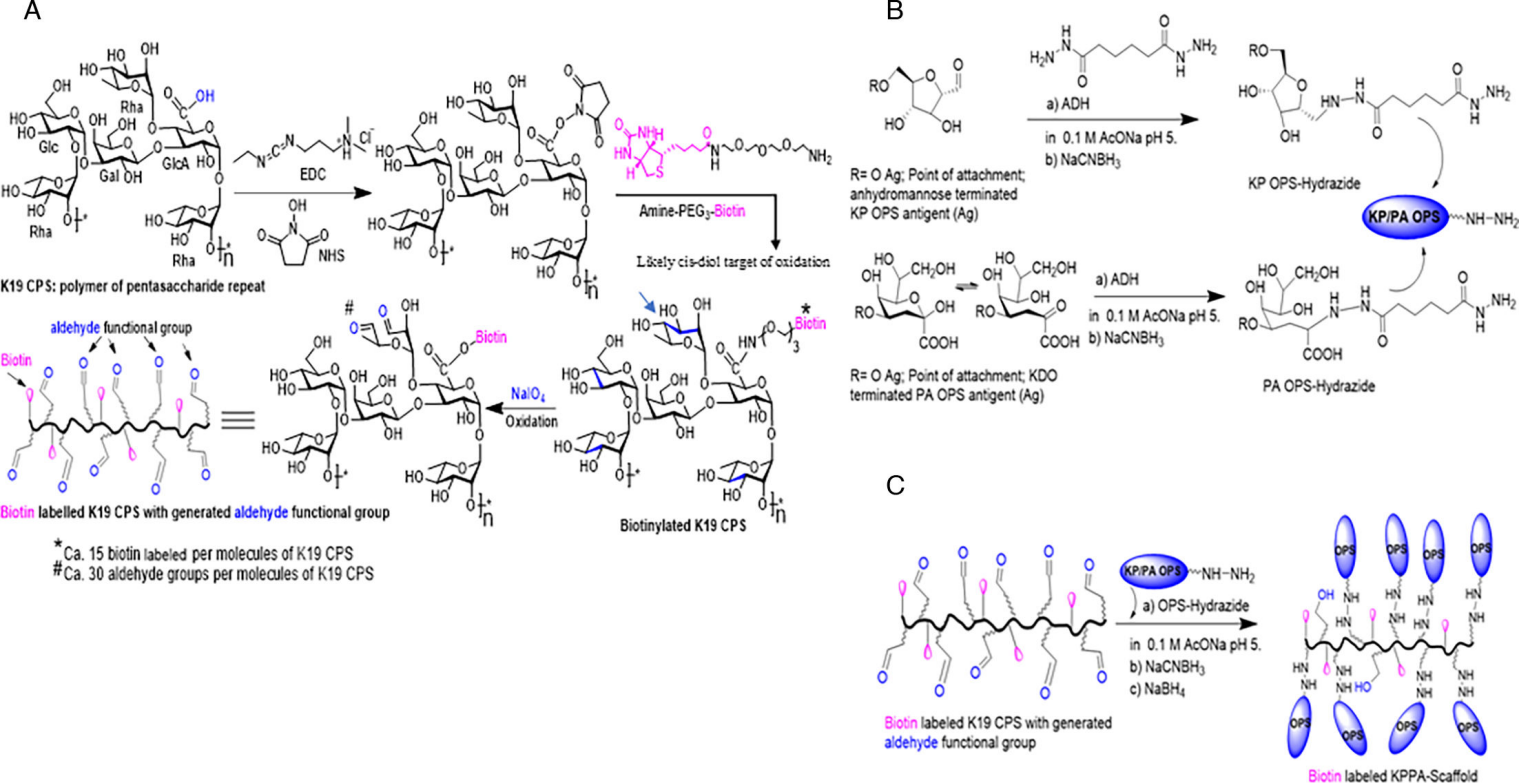
Schematic representation of the scaffolding process. (**A**) Preparation of the biotin labeling of K19 CPS with generated aldehyde functional groups. (**B**) Preparation of adipic acid dihydrazide (ADH)-labeled KP and PA-labeled OPS. (**C**) Preparation of KP/PA scaffold on modified K19 CPS backbone.

We chose *Klebsiella oxytoca* (KO) K19 CPS as the backbone polymer for constructing the scaffold because it offers several advantages. This K19 CPS is negatively charged and is made up of a hexasaccharide repeating unit containing a glucuronic acid branched with a terminal rhamnosyl residue. The chemical structure of the K19 repeating unit is shown in Fig. 1A. *Klebsiella* core-O polysaccharide/O polysaccharide (COPS/OPS) addition and MAPS complex formation lead to pronounced increases in molecular size. Furthermore, as the K19 CPS repeat unit comprises neutral and acidic monosaccharides, as well as several vicinal diols, it is amenable to many conventional chemical conjugation approaches, including carbodiimide linkage to carboxylic acids and oxidation/reductive amination approaches.

KP OPS were extracted and purified from KP reagent strains as described previously by nitrous acid deamination that cleaved the labile linkage at the outer core polysaccharide KDO, yielding liberated OPS with a reducing end 2,5-anhydromannose (4, 29) as shown in Fig. 1B. PA core and OPS (COPS) was extracted from bacterial LPS by boiling in acetic acid, which breaks the labile bond between 3-deoxy-D-manno-octulosonic acid (KDO) and lipid A (Fig. 1B). KP OPS and PA COPS were then purified essentially to homogeneity with a series of tangential flow filtration (TFF) and chromatography steps. To maximally preserve the repeat epitopes of KP OPS or PA COPS and generate well-defined scaffold constructs that comprised single K19 backbones that bore multiple OPS repeats, we devised a strategy whereby O polysaccharides were labeled at their reducing end carbonyl (PA COPS KDO ketone or KP OPS 2,5-anhydromannose aldehyde) with adipic acid dihydrazide (ADH) that installed a reactive hydrazide group (Fig. 1B). Separately, K19 CPS underwent limited biotinylation through carbodiimide-mediated coupling at the carboxyl group of the single glucuronic acid present within each repeat and limited periodate oxidation to generate approximately 15 biotin molecules and 30 aldehydes per K19 saccharide chain (Fig. 1A). Scaffold formation was accomplished by reductive amination by mixing the activated OPS-hydrazide and biotinylated-oxidized-K19, with reduction of the imine hydrazone bond with sodium cyanoborohydride, followed by reduction with sodium borohydride, especially to reduce unreacted aldehyde groups to alcohol functional groups in the K19 CPS backbone (Fig. 1C).

**Fig 2:**
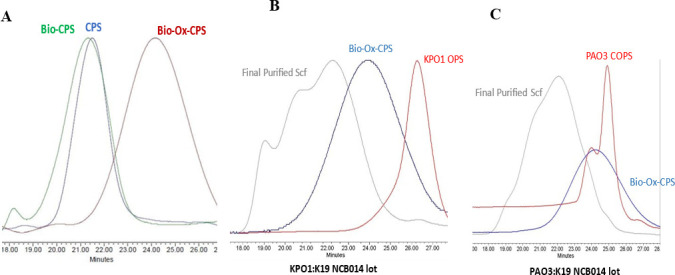
(**A**) HPLC-SEC profiles of K19 CPS (blue trace), biotinylated K19 CPS (green trace), and biotinylated-oxidized K19 CPS (red trace). Oxidation of K19 resulted in a shift to lower molecular size. (B and C) Representative scaffold HPLC chromatograms with intermediate polysaccharides OPS/COPS (red), biotinylated-oxidized K19 CPS (blue), and final purified scaffold (gray), shown for KPO1:K19 NCB014 (**B**), PAO3:K19 NCB014, and (**C**) scaffold lots. COPS/OPS:K19 scaffold formation resulted in a species larger than either component alone.

While OPS labeling was not associated with a measurable size increase, oxidation of K19 resulted in a measurable shift in apparent lower relative molecular size (Fig. 2A).

However, size-exclusion chromatography with multi-angle light scattering (SEC-MALS) analysis of oxidized and biotinylated K19 CPS (Ox-Bio-K19) and native K19 CPS indicated that the average molecular weight following biotinylation/oxidation was essentially unchanged (Table 1).

**TABLE 1:** SEC-MALS analysis of oxidized and biotinylated K19 CPS (Ox-Bio-K19) and native K19 CPS

Sample (lot number)	aMw (kDa)^*a*^	Uncertainty of MW (%)	Radius (nm)	Uncertainty of radius (%)	Percent recovery of injected mass (%)
K19 CPS (A1**-**CVD170915-01)	338.8	2.10	38.3	5.20	98.66
K19 bio, ox CPS (A1-CVD180713-04)	367.5	4.50	64.7	5.00	106.98

^*a*^ aMw, average molecular weight.

COPS/OPS:K19 scaffold formation was accompanied by a pronounced shift in molecular size by HPLC-SEC to a species that was larger than either the labeled COPS/OPS or Ox-Bio-K19 (Fig. 2B and C). We found that the overall molecular size profile of the different scaffolds largely overlapped. This notion agrees with the observed OPS:CPS ratio determined by ^1^H-NMR, which was similar among the different constructs as shown in Table S2. In addition to ^1^H-NMR, the OPS content in the scaffolds was also measured using a competitive inhibition enzyme-linked immunosorbent assayELISA (CI-ELISA) with the appropriate rabbit polyclonal anti-OPS antibody as described in the supplemental material (“Analytical testing of scaffold”).

**Fig 3:**
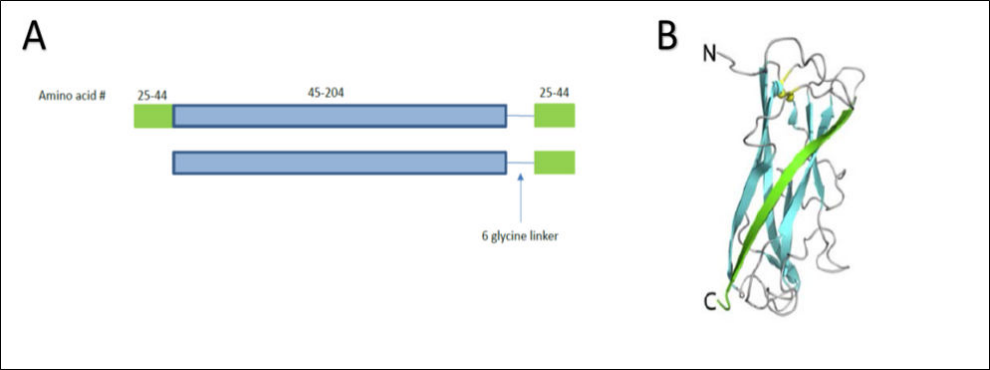
Donor strand complementation approach to produce properly folded MrkA monomers. (**A**) Strategy used for MrkA donor strand complementation. (**B**) Crystal structure of the donor strand complemented *Escherichia coli* FimA (30).

**Fig 4:**
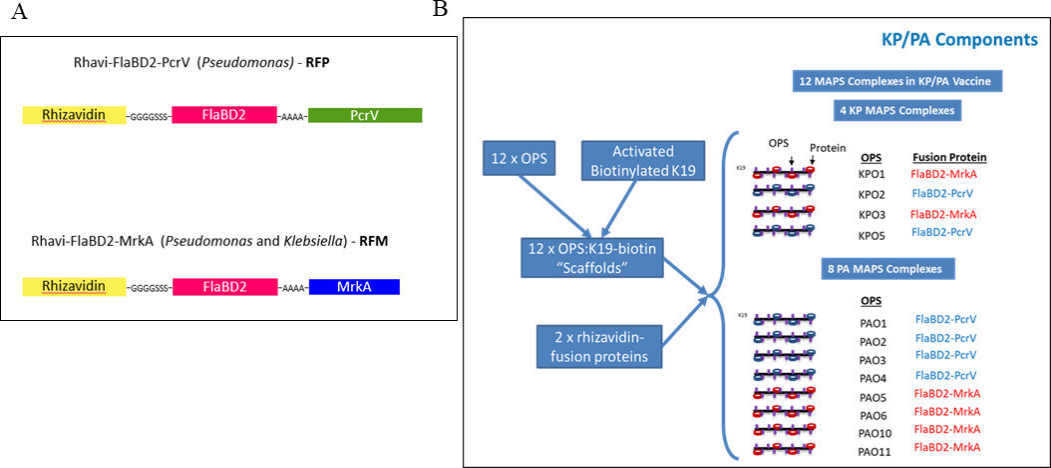
(**A**) Diagram of the two primary candidate fusion proteins selected KP and PA MAPS formulations: (upper) Rhavi-FlaB-PcrV (RFP) and (lower) Rhavi-FlaBD2-MrkA (RFM) constructs. An alanine linker separates the two pathogen-specific fusion proteins that are separated from Rhizavidin and the 6XHis tag with glycine/serine linker sequences. (**B**) General scheme for the assembly of the KP/PA components to the 12 MAPS complexes.

As fusion protein pathogen-specific sequences, we elected to use established protective antigens from both KP and PA. Each of these proteins has been used as a vaccine antigen and shown to induce protective antibodies in preclinical studies (15–17, 22). They were also good carrier proteins for polysaccharide antigens (4). For the KP component, we selected the type 3 major fimbrial protein MrkA. In order to adapt MrkA for use as a vaccine antigen, we utilized the previously described donor strand complementation approach (30), where the C-terminus of the MrkA protein was fused to a short stretch of the N-terminal MrkA sequence, thereby approximating inter-subunit packing interactions (Fig. 3). For the PA protein components, we elected to include two different protein antigens. This included PcrV, which forms the tip of the type III secretion system molecular syringe that injects cytotoxins that cause host cell death. As the second protein antigen, we selected domain 2 of B-type flagellin (FlaBD2), which lacks the Toll-like receptor 5 agonist region and forms the solvent-accessible surface of the FlaB flagellin when incorporated into the bacterial flagellum (31). These three protein antigens were used to generate two different fusion proteins with rhizavidin (Rhavi): Rhavi-FlaBD2-MrkA and Rhavi-FlaBD2-PcrV (Fig. 4A).

MAPS complexes (Fig. 4B) were formed by mixing the biotinylated scaffolds with individual fusion proteins, for which similar final protein:polysaccharide ratios were achieved in an acceptable range of 2–4:1 (wt:wt) between the different purified MAPS.

### Induction of anti-O polysaccharide and anti-fusion protein antibodies in rabbits

**Fig 5:**
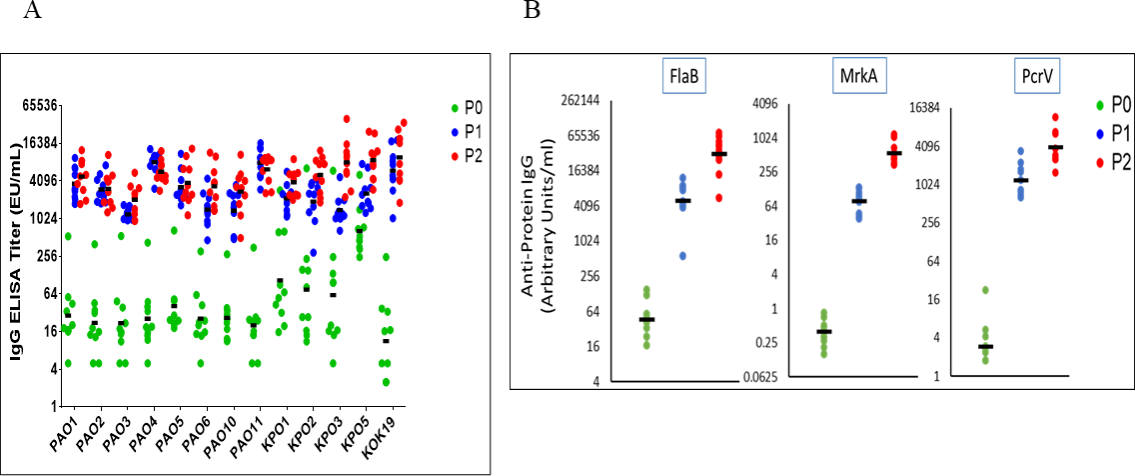
Antibody responses to O polysaccharide antigens (**A**) and pathogen-specific proteins (**B**). Rabbits (10/group) were immunized on days 0 and 28 with the KPPA MAPS vaccine. Serum was obtained on days 0 (P0), 28 (P1), and 42 (P2).

In order to assess the immunogenicity of the fully valent formulation that incorporated the 12 scaffold MAPS complexes (hereafter, “KPPA MAPS vaccine”), we immunized rabbits twice at 28 day intervals with the 12 component formulation. The vaccine was administered with aluminum phosphate adjuvant. Sera taken directly prior to the first dose (P0), 14 days after the first dose (P1), and 28 days after the second dose (P2) were assessed for IgG antibodies against the different polysaccharides and protein antigens (Fig. 5). We found that high IgG titers were induced to all 12 OPS antigens (Fig. 5A) and 3 pathogen-specific protein components (Fig. 5B). Strikingly, antibody levels to the COPS/OPS antigens were maximal in most animals after a single dose, with all animals achieving greater than fourfold seroconversion. A geometric mean-fold increase of >20 was noted for all OPS types after two doses of vaccine. High anti-K19 titers were also seen in all animals that were similar to the level seen for the OPS antigens (Fig. 5A). We also found that KPPA MAPS immunization induced high anti-protein levels, although kinetics were different from those seen against the polysaccharide antigens, with a measurable boost response occurring after the second dose (Fig. 5B) for all three proteins.

### Protection against bacterial challenge in mice after passive transfer of rabbit immune sera

**Fig 6:**
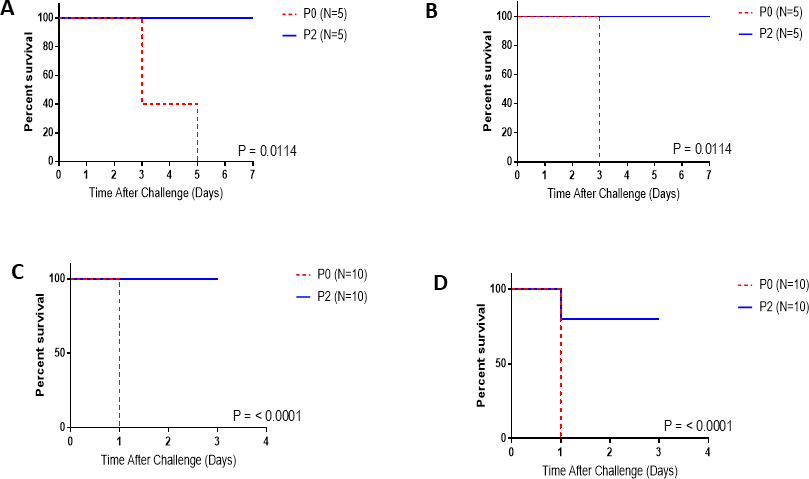
Passive protection against lethal *Klebsiella* and *Pseudomonas* infection by KPPA MAPS antisera. Representative Kaplan-Meier plots showing protection against (**A**) KP O1:K2, (**B**) KPO2:K2, (**C**) PA O6, and (**D**) PA O5.

We documented previously that passive transfer of sera raised against glycoconjugates of KP OPS linked to PA Fla proteins protected against bloodstream infections with KP (4). To confirm that antibodies raised against the polysaccharide and protein antigens would protect against disseminated infection, we utilized a mouse peritonitis model (see “Mouse passive protection from lethal sepsis” in Materials and Methods) of KP or PA challenge. In accordance with prior results seen for glycoconjugates, we found that passive transfer of sera from rabbits immunized with OPS-containing vaccines promoted robust protection against both KP and PA isolates that expressed conjugate vaccine serotype lipopolysaccharides. When compared to pre-immune sera, passive administration of post-vaccination sera significantly protected against lethal challenge with different KP strains expressing vaccine serotype OPS in the context of different capsule types. We found 100% protection against KPO1 strain B5055 (85% protection for KPO3 strain 700603 and 90% protection against KPO2 strain TPEVGH-KPN-12) (Table 2). Passive administration of the immune antisera was highly protective (*P* < 0.0001) against the four PA O serotypes tested, although the efficacy against PA O11, though significant, was less than that against the other serotypes. Representative Kaplan-Meier plots are shown for two strains of PA and two of KP (Fig. 6)

**TABLE 2:** Passive protection from lethal sepsis

Challenge strain	Survival of controls (P0) (efficacy studies)	P2-treated survival (efficacy studies)	*P*-value Fisher’s exact test (two-tailed)	Average serum IgG level in controls^*a*^ (ng/mL)	Average serum IgG level in P2^*a*^ (ng/mL)
KPO1	0/20 (0%)	20/20 (100%)	<0.0001	<15	160
KPO2	0/20 (0%)	18/20 (90%)	<0.0001	<15	628
KPO3	1/20 (5%)	17/20 (85%)	<0.0001	19	415
PA O4	2/20 (10%)	19/20 (95%)	<0.0001	<15	402
PA O5	0/20 (0%)	19/20 (95%)	<0.0001	<15	195
PA O6	1/20 (5%)	18/20 (90%)	<0.0001	<15	332
PA O11	0/20 (0%)	8/20 (40%)	0.003	<15	292

^*a*^ Collected at the time of infectious challenge (0 h) after 0.2 mL IP administration of antisera at −20 and −2 h. PA challenges at 3 × 10^7^ CFU. KP O1 and KP O2 at 2 × 10^4^ CFU and KP O3 at 1 × 10^5^ CFU.

### Active immunization

**Fig 7:**
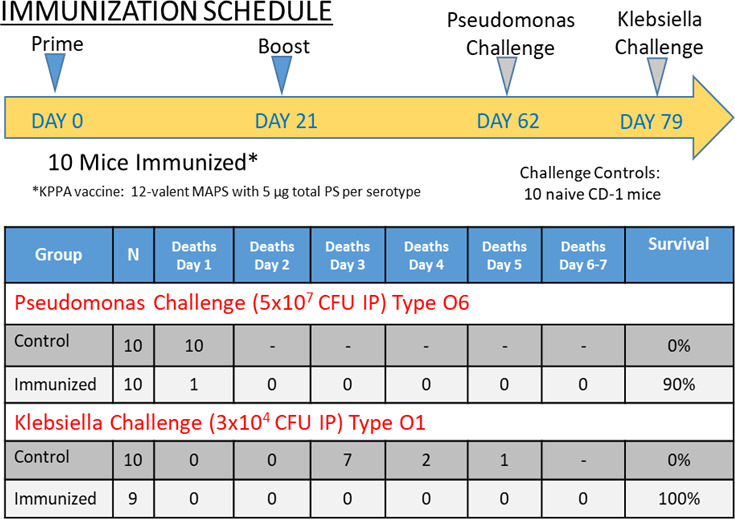
Active immunization with KPPA MAPS vaccine protects against lethal KP and PA infection. CD-1 mice (10/group) were immunized on days 0 and 21 with 12-valent KPPA MAPS (5 µg total PS/serotype). Immunized mice and naive CD-1 controls were challenged on day 62 with *Pseudomonas aeruginosa*. Survivors of the *Pseudomonas* challenge were challenged 17 days later (day 79) with *Klebsiella* O1:K2.

Mice immunized with the 12-valent KPPA MAPS vaccine were protected against challenge at 41 days after the last immunization with a PA O6 strain SBI-N (FlaA2) (9/10 survived vs 0/10 controls), and the same mice that survived the PA challenge were later challenged at day 79 (i.e., 38 days after the PA challenge) with KPO1 strain B5055 and protected (9/9 mice survived vs 0/10 of non-randomized control mice) (Fig. 7).

### Functional activity of vaccine-induced antibodies

Functional antibody responses against the different vaccine antigens are presumed to be active via different mechanisms. Antibodies directed against the protein antigens are expected to be function blocking, either by inhibition of KP adhesion (MrkA), PA motility (FlaB), or PA host cell intoxication (PcrV). Accordingly, we assessed the vaccine-induced antibodies with *in vitro* functional assays designed to measure each of these parameters.

#### Adherence

**Fig 8:**
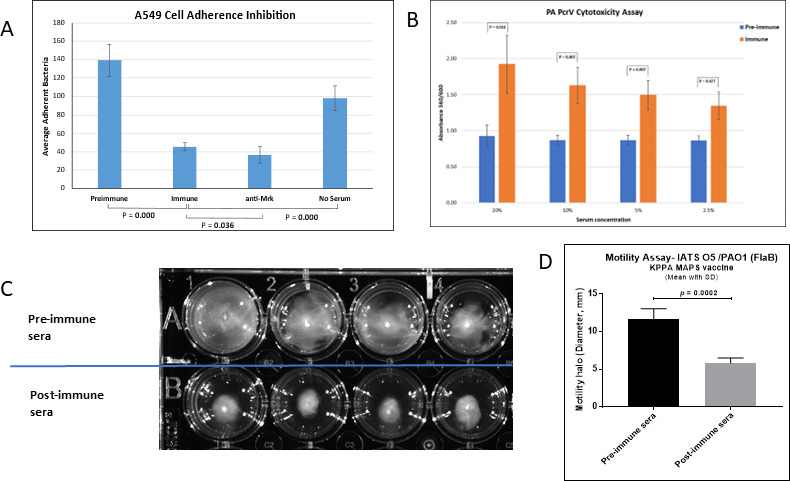
Functional activity of preimmune and immune sera generated in rabbits by pathogen-relevant fusion proteins in 12-valent KPPA MAPS vaccine. (**A**) MrkA antibody function. Cells: A549 lung epithelial-derived tissue culture; bacteria: *Klebsiella* strain 1015 O4:K15 (not in vaccine). (**B**) PcrV antibody function*. Pseudomonas* injection of cytotoxins through T3SS induces apoptosis of lung epithelial cells, as indicated by loss of Alamar Blue dye absorbance. Cells: A549 lung-epithelial-derived tissue culture; bacteria: *Pseudomonas* strain PAK (known to express PcrV and cytotoxins). (**C**) FlaB antibody function. Soft agar motility inhibition assay. Bacteria: *Pseudomonas* FlaB+ strain PAO1 was added in quadruplicate to soft agar containing either preimmune or KPPA MAPS post-immune sera, and (**D**) the diameter of PA migration from the central stab was measured.

**Fig 9:**
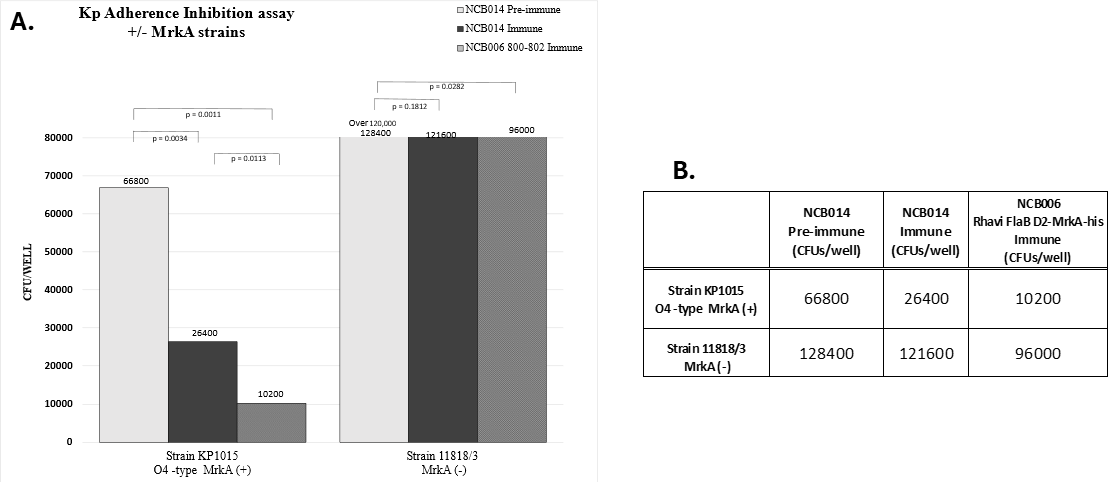
MAPS-elicited antisera inhibit adherence of MrkA+ KP to A549 lung epithelial cells but not of MrkA− KP. (**A**) Rabbits were immunized with NCB014 or with Rhavi FlaB D2-MrkA-his alone on a K19 backbone, and the sera were tested for the ability to inhibit the adherence of a MrkA+ (KP1015) or a MrkA− KP strain (strain 11818/3) to A549 cells. The MrkA-immune sera (NCB014), but not the preimmune (P0), reduced the adherence of the MrkA+, but not that of the MrkA− KP strain, similar to the inhibition with the MrkA-only antisera. (**B**) Neither NCB014 immune nor MrkA-specific antisera were able to inhibit the binding of a MrkA− KP strain.

We found that post-vaccination sera strongly inhibited the ability of a KP strain with an O4 OPS serotype (i.e., non-vaccine OPS serotype) to adhere to A549 lung epithelial cells. This strain was included to confirm that inhibition was not due to anti-OPS antibodies (Fig. 8A). We also observed that anti-MrkA sera generated with a vaccine formulation in which Rhavi FlaB D2-MrkA-his alone was expressed on the K19 CPS backbone inhibited adherence of a MrkA^+^ KP strain (Fig. 9A), but not that of a KP strain that did not express MrkA (Fig. 9B). We screened multiple KP isolates obtained from humans with bacteremia from different countries for the presence of MrkA. MrkA was detected in 12 of the 14 isolates with OPS types not included in the vaccine when assayed by three different methods (Table 3). Thus, functionally active antibodies to MrkA may expand the coverage of this vaccine to KP strains with an OPS not included in this vaccine.

**TABLE 3:** MrkA expands coverage to *Klebsiella* strains expressing non-vaccine OPS types

Strain ID	Country of origin	O type	Bacterial agglutination	Bacterial binding flow cytometry	MrkA gene presence (PCR)
70721	USA	Not (O1, O2, O3, O5)	No	No	No
GN05275	USA	Not (O1, O2, O3, O5)	Yes	Yes	Yes
NUH5575	Japan	Not (O1, O2, O3, O5)	Yes	Yes	Yes
170241	USA	Not (O1, O2, O3, O5)	Yes	Yes	Yes
60111	USA	Not (O1, O2, O3, O5)	Yes	Yes	Yes
5205	South Africa	Not (O1, O2, O3, O5)	Yes	Yes	Yes
7075	South Africa	Not (O1, O2, O3, O5)	Yes	Yes	Yes
7069	South Africa	Not (O1, O2, O3, O5)	Yes	Yes	Yes
11818/3	Mali	Not (O1, O2, O3, O5)	No	No	No
9536/15	Mali	Not (O1, O2, O3, O5)	Yes	Yes	Yes
12287/3	Mali	Not (O1, O2, O3, O5)	Yes	Yes	Yes
KP1015	Denmark	O4	Yes	Yes	Yes
NUH5218	Japan	O4	Yes	Yes	Yes
EC1793	Singapore	O4	Yes	Yes	Yes

#### Flagellin-mediated motility and PcrV-mediated cytotoxicity

Sera from rabbits immunized with the scaffold-MAPS vaccine also demonstrated functional activity against the PA protein antigens. Among the various toxins expressed by PA, direct injection of cytotoxins ExoU and/or ExoS after contact-dependent secretion through the PcrV tip translocator on the type III secretion system has been shown as a major contributing factor to PA virulence (16, 17). We found that post-vaccination sera from rabbits immunized with MAPS formulated with fusion proteins that incorporated the PcrV antigen markedly impaired the ability of PA PAK to intoxicate A549 lung epithelial cells, with significantly less cell cytotoxicity seen after incubation with post-vaccination sera compared to paired pre-immune sera (Fig. 8B). We found that post-vaccination sera significantly inhibited the motility of different FlaB-expressing PA isolates, including a commonly used O5 research isolate (PAO1), as well as clinical isolates that expressed the FlaB flagellin and OPS serotypes included in the vaccine or were of a heterologous non-vaccine OPS serotype (Fig. 8C).

#### Vaccine-induced antibodies mediate opsonophagocytic activity

**Fig 10:**
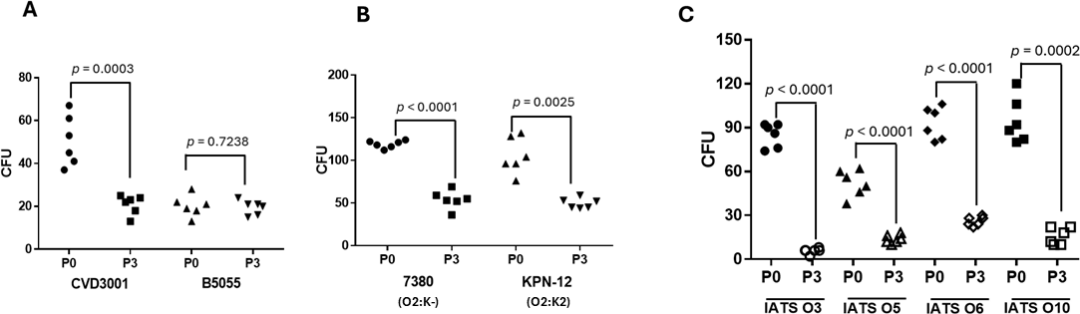
Compared to preimmune (P0) sera, antisera elicited by the NCB014 MAPS vaccine (P3) mediated the killing of KP and PA in a RAW 264.7 opsonophagocytic assay. NCB014-immune sera did not kill the KP O1:K2-encapsulated strain (B5055) but did kill its unencapsulated mutant (CVD3001). (**A**). However, NCB014-immune sera killed both an unencapsulated (7380) and a K2-encapsulated KP O2 strain (**B**). NCB014-immune sera mediated the killing of four different PA serotypes (**C**) The immune sera also killed the remaining four serotypes in the MAPS vaccine (data not shown).

The ability of MAPS-induced antibodies to mediate the uptake and killing of PA and KP serotypes included in the MAPS vaccine was tested by an assay using the RAW cell 264.7 mouse macrophage-like cell line (see Materials and Methods). When compared to pre-immunization sera, immune sera promoted the uptake and killing of multiple KP and PA target strains (Fig. 10). While the immune sera did not promote the killing of KP strain B5055 (O1:K2), there was robust killing of its unencapsulated mutant, CVD 3001 (Fig. 10A). The presence of CPS reduced the ability of immune sera to kill KP strains of the same serotype, although the immune sera was highly protective in *in vivo* experiments (see Fig. 7 and 10A). In contrast, immune sera were able to mediate the killing of both an unencapsulated strain of KP O2 (7380) and a K2-encapsulated strain of KO O2 (Fig. 10B). All four IATS O types of PA tested were killed in the presence of immune sera (Fig. 10C). We did not observe any bactericidal activity with the immune sera (data not shown).

## DISCUSSION

In recent reports, the CDC identified KP and PA as “urgent” and “serious” threats to public health, in large part due to their dramatic increase in antimicrobial resistance (32). Deaths from antibiotic-resistant bacterial infections are projected to increase from 700,000 deaths globally each year today to 10 million deaths annually by 2050, surpassing all other causes of mortality (33, 34). These observations have led to a renewed effort to rejuvenate a stagnated antibiotic development pipeline to include public support to make the enterprise profitable. They have also led to greater efforts to support antibiotic stewardship programs. To date, neither effort appears to have had a major impact on the prevalence of antimicrobial resistance. Indeed, the COVID pandemic has led to increased antimicrobial usage by clinicians and resistance (35). As an additional approach, the development of vaccines against these bacterial pathogens might reduce the acquisition and spread of KP and PA, prevent infections, and thereby reduce the need for antibiotic use (36, 37).

As public health tools, licensed vaccines have a proven track record for counteracting AMR among bacterial pathogens, and the development of vaccines for new and emerging AMR threats represents an important priority. Earlier, a 24-valent KP capsular polysaccharide vaccine and an 8-valent PA vaccine in which the OPS was conjugated to recombinant PA exoprotein A were individually tested in early-stage clinical studies (5, 13). Antisera raised to the two vaccines were made into a hyperimmune immunoglobulin for intravenous use (H-IVIG). A single infusion of this H-IVIG was tested for the prevention of KP and PA HAIs among acute care patients in ICU settings. While *post hoc* analysis suggested evidence of protection at early time points following antibody infusion, the decreasing antibody levels during the entire observation period were unable to prevent infections at later stages of the clinical trial (38). Despite their long history of causing nosocomial infections, further efforts toward the development of KP and PA vaccines (with the exception of vaccines targeting cystic fibrosis patients and burn patients [39, 40]) were not prioritized due to the efficacy of antibiotic treatment at the time (41), although a recent clinical trial with a *Pseudomonas* outer membrane recombinant fusion protein OprF/l vaccine was conducted in ICU patients on ventilators with no effect on infection rates (14).

Since the earlier 24-valent KP capsular polysaccharide vaccine advanced to clinical development (5, 18), Trautmann and others (19) demonstrated that four KP OPS serotypes would cover ~80% of clinical isolates. Consequently, KP vaccine development efforts now focus on targeting the KP O polysaccharide and include bioconjugates (20), O polysaccharide/flagellin conjugates (4), as well as semi-synthetic polysaccharide conjugates (42).

Our current vaccine strategy combines OPS antigens and protective surface proteins from both *Pseudomonas* and *Klebsiella* using MAPS, a vaccine platform that is being evaluated clinically with a multivalent pneumococcal vaccine in adults and children and shown to be safe and immunogenic (27, 43). We developed a scaffold MAPS approach whereby multiple copies of long-chain O polysaccharides are linked to a large polysaccharide backbone (K19 CPS) to which pathogen-specific proteins are added to induce functionally relevant anti-protein antibodies. Earlier studies with the MAPS platform generated Th1 and Th17 responses (27, 44). Pathogen-specific proteins from *Klebsiella* (i.e., MrkA) and *Pseudomonas aeruginosa* (PcrV and flagellin) were found to induce functionally active antibodies that may play a role in protection against KP and PA infection and generate Th1 and Th17 responses.

We used aluminum phosphate (AlPO_4_) (AdjuPhosR, Brenntag Biosector, Frederikssund, Denmark) as an adjuvant for the MAPS vaccine. Most of the molecular mechanisms for alum were based on studies with aluminum hydroxide [Al(OH)_3_]. While there has not been a clear rationale for choosing between the two adjuvants, Al(OH)_3_, with its high isoelectric point, adsorbs antigens more efficiently than AlPO_4_, which has a neutral or slightly acidic isoelectric point. The positively charged Al(OH)_3_ allows for stronger interaction with the negatively charged cell membrane than the negatively charged AlPO_4_. A recent comparison of the effects of Al(OH)_3_ and AlPO_4_ using flow cytometry *in vivo* with mice and quantitative mass spectrometry-based proteomics *in vitro* with human primary monocytes did find that the two adjuvants acted differently on the innate immune system. They attracted different cell types [neutrophils for Al(OH)_3_ vs monocyte macrophages for AlPO_4_] to the site of injection and differed in inducing immune system-related processes (45). The backbone MAPS approach has several advantages over conventional glycoconjugates. Lattice conjugates that are formed by multipoint linking across the OPS chain can achieve high molecular weight; however, this is accompanied by potential loss of immunogenic epitopes by steric mechanisms (46, 47). In contrast, the scaffold approach incorporates multiple copies of long-chain O polysaccharides with full OPS epitope preservation, conferring upon the OPS hapten the large size of capsular polysaccharides. Moreover, this approach incorporates into a single molecule multiple OPS non-reducing terminal ends that are the sites of functionally dominant epitopes for some polysaccharides and represent the most solvent-accessible OPS epitopes on the bacteria, particularly when terminated with a novel motif (e.g., methylated mannose cap residues of *Klebsiella* OPS types O3 and O5 [30]). Another advantageous feature of the MAPS construct is the defined single point of complex formation between the rhizavidin fusion protein and the scaffold backbone biotin (with a dissociation constant of 10^−14^), which permits full preservation of protective B- and T-cell fusion protein epitopes (Fig. 1, schema 1C). In contrast, conventional conjugates are formed by cross-linkage at random sites of the protein, which may reduce the concentrations of B cell and T cell epitopes. Hence, our isolated antigen components are specifically reassembled into an integrated macromolecular complex, mimicking some chemical and physical features of a whole-cell construct. Such a complex could lead to the activation of more comprehensive B- and T-cell immune responses and thereby provide the multipronged protection characteristic of whole cell vaccines (25).

This KPPA MAPS vaccine has several advantages over other formulations. For example, since KP LPS is relatively small, vaccines that have one to two KP OPS molecules are poorly immunogenic. The KPPA MAPS vaccine has multiple copies of each OPS, which facilitates a robust anti-KP OPS antibody response. Moreover, the vaccine includes MrkA as a carrier protein, a type III fimbriae used by KP to attach to cells. Antibodies to MrkA inhibit KP attachment and protect against lethal KP infection (22). Since >90% of KP strains express MrkA, inclusion of this protein in the MAPS broadens the coverage against KP strains. The MAPS vaccine is highly effective preclinically in preventing PA infections. In addition to the inclusion of PA OPS, the MAPS vaccine includes two virulence factors, PcrV and flagellin, each of which has been shown to have protective efficacy in animal models (16, 17). The PcrV type 3 secretion system apparatus is used by PA to inject cytotoxins into host cells, while antibodies to flagellin inhibit PA motility, essential to PA virulence. Thus, in addition to the protective efficacy of anti-OPS antibodies to KP and PA, which are the basis of other vaccine formulations, the KPPA MAPS vaccine includes pathogen-relevant proteins as both carrier proteins and as antigens that increase the vaccine coverage beyond the OPS types. We are unaware of any other vaccine that simultaneously targets both PA and KP, which are considered urgent (KP) and serious (PA) threats.

We found that rabbits immunized with the KPPA MAPS vaccine achieved high antibody levels to all of the vaccine polysaccharide and protein antigens, unlike the immune responses to some vaccine components that may have been adversely impacted in a multivalent formulation (48, 49).

KPPA MAPS vaccine-induced antibodies protected mice against lethal *Klebsiella* and *Pseudomonas* infection (Table 3), but the antibody levels associated with the protection against lethal peritonitis are preliminary and need to be confirmed in additional animal models.

In the case of KP, there has been discussion in the literature whether antibodies to subcapsular antigens, such as O antigens, can access their targets (7, 50), particularly for certain capsular types such as K1 and K2. This is not a concern for the acapsular *Pseudomonas*. We selected the pathogen-specific proteins MrkA, PcrV, and flagellin, based on published reports that antibodies to each of these virulence factors are protective in animal models of lethal infection (51–55). We demonstrated that these proteins induced a strong antibody response, even after the first dose, and these antibodies were functionally active using *in vitro* assays. KPPA MAPS vaccine-induced antibodies to the type 3 KP fimbriae, MrkA, reduced the ability of *Klebsiella* to adhere to the human lung epithelial cell line, A549, while the same antisera containing antibodies to PcrV, the type 3 secretion system of *Pseudomonas*, were able to protect the same A549 cell line from PA cytotoxins. MAPS-induced antibodies that had high levels of antibodies to FlaBD2 markedly reduced the flagellin-dependent motility of *Pseudomonas*. Each of these three proteins is widely expressed on most KP (MrkA) and PA (FlaB and PcrV) clinical isolates (6, 12). While these proteins expand the coverage of the KPPA MAPS vaccine to non-vaccine O serotypes that may be encountered by simultaneously blocking multiple virulence factors and promoting immune clearance, we are unable to parse out the contributions of these antigens from the OPS antigens. It may be possible to assess the protective activity of OPS or the pathogen-specific proteins alone by evaluating monocomponent vaccines. Such studies could also provide information on whether there are any synergistic protective activities that may occur with the combined constructs. It is worth noting that this is the first instance of using MAPS to include antigens across more than one specific pathogen.

Unlike the case for “public health vaccines” such as those for COVID, measles, and polio that are widely administered to the population, the optimal target population profile for this, and indeed any vaccine developed for healthcare-associated infections, is difficult to precisely define. Certainly, patient populations at higher risk of developing infections with AMR bacteria should be considered as a primary target group. This would include patients undergoing elective high-risk surgery or admission to a long-term acute care (LTACH) facility. Extensive studies by Elizaga et al. (56) have shown that patients in LTACH are highly likely to be colonized with gram-negative bacteria and at risk for subsequent sepsis and transport to the hospital (57). The vaccine could also be more narrowly targeted to otherwise healthy individuals undergoing procedures (e.g., orthopedic procedures) that would put them at risk of acquiring infection or to patients at discharge from the hospital since such patients are likely to be re-admitted within a year of discharge (58). Very ill patients at risk of infection with AMR bacteria respond to immunization and therefore might be considered for immunization. For example, patients admitted to the ICU respond to a *Pseudomonas* vaccine (14, 59) as do patients admitted for acute trauma (60). In the latter study, robust antibody levels were observed at the first time point, 14 days after immunization. The response at earlier time points needs to be determined. Furthermore, groups at occupational risk of emergency surgery or burn wound injury (e.g., military, firefighters, and police) may benefit from such immunization. Finally, if proven safe and effective, it might be used for all older adults at the time they receive other vaccines (e.g., pneumococcal, shingles, and influenza) since these individuals are most at risk of acquiring these infections. Since any clinical efficacy trial for healthcare-associated vaccines would require a very large, expensive study, it is more likely that “proof” of efficacy would depend on finding correlates of protection, as is being considered for other vaccines (e.g., COVID-19 and group B streptococcal and pneumococcal vaccines) (61–63).

Our study has a few limitations. While the MAPS KPPA vaccine was sufficiently immunogenic in both rabbits and mice to provide protective levels of anti-KP and anti-PA antibodies, we do not yet know if these responses will predict immunogenicity and protection in humans. Furthermore, we did not determine the relative contributions of the antibodies to the O polysaccharides or the pathogen-relevant fusion proteins (MrkA, PcrV, and flagellin) to protection in the murine infection models. As a result, although we demonstrated robust protection with the MAPS-induced antisera, we did not determine a correlate of protection from these studies. As will be the case for other vaccines that target health-care-associated infections, the greatest challenge will be to perform adequately powered clinical trials to demonstrate protection by the KPPA MAPS vaccine against KP and PA disease. Since KP and PA infections comprise only a portion of the fewer than 5% of hospitalized patients found to have nosocomial infection in a point-prevalence study (1), a clinical trial would require a large number of patients and multiple clinical trial sites. Alternatively, it may be possible to establish a correlate of protection for a KPPA vaccine in animal models, obtain marketing authorization, and then confirm protection in humans post-licensure.

## MATERIALS AND METHODS

### Bacterial strains, media, and growth conditions

The *Pseudomonas* and *Klebsiella* strains used in this study are detailed in Table S1. For routine maintenance, they were grown at 37°C in animal product-free Hy-Soy (HS) bacteriological culture media or on HS solid agar as described in supplemental material. The growth of KP and PA in fermentation culture for the production of purified polysaccharides was accomplished with fully chemically defined media supplemented with trace vitamins and elements as described in supplemental material with the following modifications: CVD 3001 culture media were supplemented with 0.004%–0.025% guanine (Sigma, MA), and PA was supplemented to 0.13 g/L with a mixture of essential amino acids (L-alanine, L-arginine, L-asparagine, L-aspartic acid, L-cysteine, L-cystine, L-glutamic acid, L-glutamine, glycine, L-histidine, trans-4-hydroxyl-L-proline, L-isoleucine, L-leucine, L-lysine, L-methionine, L-phenylalanine, L-proline, L-serine, L-threonine, L-tryptophan, L-tyrosine, and L-valine; Sigma, MA, USA). *Escherichia coli* strains expressing the recombinant rhizavidin fusion proteins were grown in Luria-Bertani medium containing carbenicillin (Carb+) at 37°C.

### Preparation of the vaccine

A detailed description of the multivalent *Klebsiella pneumoniae/Pseudomonas aeruginosa* MAPS vaccine may be found in the supplemental material. Briefly, following the growth of reagent strains in 8 L fermenters, the KP OPS and PA core and OPS (COPS) were extracted, purified, and characterized from the respective LPS from each of the reagent strains. Expression plasmids were designed and engineered to direct *E. coli* expression of triple protein fusions that encoded rhizavidin, a protein in the streptavidin family, the D2 segment of PA flagellin B, and either KP type III protein, MrkA, or the PA type III secretion protein, PcrV. The plasmids encoding the rhizavidin fusion protein were transformed into Origami B (DE3) *E. coli* (EMD Millipore), the transformed bacteria were grown in large volumes with IPTG for protein induction, which were then purified and analyzed by SDS-PAGE and BCA protein assay kit. The KP-OPS and PA-COPS were labeled at their reducing end with ADH, assayed for total carbohydrate by resorcinol assay, and for size by SEC-HPLC. K19 capsular polysaccharide was purified from a strain of *K. oxytoca* and derivatized with biotin to be used as a backbone for the KP and PA polysaccharides and the rhizavidin fusion proteins. Following labeling the KP-OPS and PA-COPS with ADH, the K19 CPS-biotin was subjected to a limited periodate oxidation in preparation for the backbone polymer assembly. The concentrated Bio-Ox-CPS and OPS/COPS-AH solutions were combined, mixed under gentle stirring overnight at room temperature, followed by adding a NaBH_3_CN solution on days 2 and 3. On day 4, the reaction mixture was diluted with 0.1 M NaOAc, pH 5.0, and NaBH_4_ was added to cap the residual unreacted aldehydes. The crude scaffold was purified by TFF microfiltration with collection of the permeate that was later concentrated and aliquoted into sterile conical tubes and stored at −20°C. The molecular size distribution of the OPS K19 scaffold was determined by HPLC-SEC. A biotin assay determined the level of biotinylation in the final purified scaffold. A CI-ELISA was performed to determine the final concentration of the OPS in the scaffold and assess their antigenicity. The OPS to K19 CPS output ratios in the purified scaffold were determined by 1H-NMR spectroscopy. (64). Total carbohydrate in the scaffold was determined by the resorcinol assay with purified individual OPS/CPS standards. MAPS complexes were generated by adding rhizavidin fusion protein to the biotinylated polysaccharide scaffold, incubating overnight, and then centrifuging to remove any insoluble material. The MAPS complexes were purified with size exclusion chromatography, with peak fractions analyzed for protein content with SDS-PAGE and BCA assay, and competitive inhibition ELISA for polysaccharide.

### Rabbit immunization

Prior to immunization of rabbits, the MAPS complexes were formulated with aluminum phosphate adjuvant approximately 48 h prior to injection. MAPS were adsorbed to adjuvant at a final concentration of 10 µg/mL of each serotype PS as a multivalent mixture with 40 mM histidine, pH 5.5, 150 mM NaCl, and 1.25 mg/mL elemental aluminum from aluminum phosphate gel with end-over-end mixing overnight at 4°C. This formulated mixture was used directly for immunizations at a volume of 0.5 mL for a 5 µg dose of each PS. Four-month-old New Zealand White rabbits (Cocalico Biologicals, *n* = 10 per group) were immunized by IM injection in the hind quarters at 0 and 28 days with 0.5 mL of the MAPS vaccine formulation suspended in sterile PBS, pH 7.4. Sera were obtained prior to the first dose (P0), 28 days after the first dose (P1), and 14 days after the second immunization (P2) and were stored at −80°C until use. Pooled serum samples were aliquoted into volumes of 2.5 mL, sterile filtered through a 0.22 μm membrane (Millipore), then stored frozen at −80°C until use.

### Mouse passive protection from lethal sepsis (mouse peritonitis model)

Outbred CD-1 mice from Charles River (6–8-week-old females) were injected IP with 0.2 mL pooled rabbit serum first at 20 hours prior to infection and then again 2 hours prior to infection (0.4 mL total). Ten mice/group received either pre-immune P0 or immune serum in replicate experiments. Bacteria were grown overnight in Hy-soy broth culture at 37°C in a shaking incubator, then sub-cultured into fresh broth and grown to mid-log phase. Bacteria were pelleted by centrifugation, then suspended in PBS to an optical density at 600 nm (OD600) of 0.3, which had been determined to correspond with approximately 10^8^ bacteria/mL. For Klebsiella strains, the bacteria were diluted in PBS to give a predetermined challenge dose of 2 × LD50 in a volume of 0.1 mL. Bacteria (0.1 mL/mouse) were injected IP, and animals were monitored twice daily for morbidity /mortality until completion of the experiment. Bacteria (dose) tested in this assay were *P. aeruginosa* strains 1071 (3 × 10^7^), SBI-N (3 × 10^7^), M-2 (2 × 10^7^), and 15AP500428 (3 × 10^7^) and *K. pneumoniae* strains B5055 (1 × 10^4^), TPEVGH-KPN-12 (1 × 10^5^), and 700603 (2 × 10^4^).

### Mouse active protection from lethal sepsis

Outbred CD-1 mice from Charles River (6–8-week-old females) were injected IP with the 12-valent MAPS vaccine at 5 mcg per serotype on days 0 and 21. Naive and immunized mice were challenged IP on day 62 with PA type O6 at 5 × 10^7^ CFU and followed for survival. Mice surviving the PA challenge were then given *Klebsiella pneumoniae* O1:K2 (strain B5055) on day 79, and these mice were followed for survival.

### Enzyme-linked immunosorbent assay (ELISA)

Anti-protein and polysaccharide IgG antibodies in rabbit sera were detected by ELISA and reported as end-point ELISA unit titers (EU/mL), essentially as reported previously. Isolated KP OPS generated as conjugates with human serum albumin (HSA) were used as coating antigen. Isolated PA COPS were used as direct coating antigen without coupling to HSA. HSA conjugates were generated by reductive amination of purified partially periodate-oxidized KP ox-OPS as described above with HSA for 3 days at room temperature, followed by the addition of 200 mg/mL sodium cyanoborohydride for an additional 18–24 hours, at which point the OPS-HSA was purified by dialysis with 3 kDa cassettes (Thermo, MA, USA) with DI water. As coating antigens for the protein antigens, full length FlaB (4), donor strand complemented MrkA, or PrcV generated as recombinant proteins with 6XHis tags, and purified by immobilized metal affinity chromatography (IMAC) were used to coat clear round-bottom nonsterile 96-well plates (BioOne, Greiner). Plates were coated with 100 µL/well in PBS, pH 7.4, of rFlaB at 1.0 µg/mL, rMrkA at 1.0 µg/mL, or 1.0 µg/mL rPcrV. Plates were incubated for 3 h at 37°C followed by six washes with PBS + 0.05 % Tween 20 (PBS-T), and were then blocked with PBS, pH 7.4, with 10% non-fat dry milk overnight at 4°C. Rabbit serum samples diluted in PBS-T + 10% non-fat dry milk, pH 7.4, were added in duplicate wells and incubated for 1 h at 37°C, followed by six washes with PBS-T. Bound rabbit IgG was detected with HRP-labeled Goat anti-Rabbit IgG (Life Technologies, CA, USA) diluted 1:2,000 in 10% non-fat dry milk in PBS-T, pH 7.4, for 1 h at 37°C. After washing, substrate (3,3′,5,5′-tetramethylbenzidine, Seracare, MD, USA) was added, and the plates were incubated on a rocker at ambient temperature for 15 min in darkness. The reaction was stopped with the addition of 1 M H3PO4, and the absorbance at 450 nm was recorded using a Multiskan FCTM Microplate Reader (Thermo, MA, USA). Test and control sera were run in duplicate. Titers were calculated by interpolation of absorbance values of test samples into the linear regression curve of a calibrated control (reference serum). The endpoint titers reported as ELISA units (EU) represent the inverse of the serum dilution that produces an absorbance value of 0.2 above the blank.

### Opsonophagocytic assay

The mouse macrophage-like cell line, RAW 264.7, originally obtained from ATCC, was maintained in culture in Dulbecco’s modified Eagle’s medium (Gibco), fetal bovine serum, and penicillin/streptomycin for up to 50 passages until discarded for a new aliquot. On the day before the assay, the RAW cells were counted, viability determined by trypan blue dye exclusion, and seeded into a 96-well culture plate (Corning, Kennebunk, ME, USA) at 2 × 10^5^ cells/well and incubated overnight at 37°C in 5% CO_2_. Also on the day preceding the assay, bacteria were inoculated from frozen stocks into 2 mL of Hy-soy broth and grown overnight at 37°C in a shaking incubator. One the following day, 1 mL of the overnight culture was inoculated into a 50 mL plastic tube containing Hy-Soy broth and grown to early log phase (OD_600_ = 0.3). Bacteria were then washed one time in PBS and resuspended to an OD_600_ of 0.3, which corresponds to a bacterial concentration of 2 × 10^8^ CFU/mL. Bacteria (1:10,000 dilution) were mixed with various concentrations of serum diluted in PBS-1% bovine serum albumin (BSA) and incubated at 37°C for 30 min. Growth media were removed from the RAW cells, which were then washed with PBS. After the wash was removed from the cells, the bacteria and serum mixture was added in duplicate wells with RAW cells at an MOI (RAW cells to bacteria) of 100. An aliquot of 10 µL was immediately taken, diluted in PBS-1% BSA, and plated on TS agar plates for the determination of the starting bacterial concentration. The microtiter plate was placed on a shaker in a 37°C incubator and rotated at the 150 rpm setting for 3 h. At 3 h, samples were taken from each well, diluted in PBS-1% BSA, and plated onto to agar plates (supernatant counts). The remaining supernatants were removed, and cells were washed with PBS-1% BSA. Following the removal of wash fluid, distilled water was added to each well to lyse the cells. At the end of 30 min at 400 rpm at 37°C, cells were visually inspected for lysis, the wells were mixed by pipetting, lysates were spotted in triplicate onto agar plates for CFU determination, and incubated overnight at 37°C. Colonies were then visually counted.

### Tissue culture

Cells were sub-cultured by scrapping and passage at a ratio of 1:4 into fresh medium every 3 or 4 days. Human lung epithelium-derived A549 cells (ATCC CRM-CCL-185) were grown at 37°C and 5% CO_2_ in F-12K nutrient medium with L-glutamine supplemented with 10% FBS and penicillin/streptomycin antibiotic. Cells were sub-cultured by treatment with Versene cell detachment solution and passage at a ratio of 1:4 into fresh medium every 3 or 4 days.

### Motility inhibition assay

The swimming motility assay was prepared by pouring 1 mL of motility agar media (1% tryptone, 0.5% NaCl, and 0.3% agar) per well into a sterile 24-well plate containing 50 μL of neat pre- and post-immune sera in duplicate with mixing by pipette. Overnight static cultures were grown in HS media at 37°C, washed in PBS twice, and normalized to an OD_600_ of 1.0. Bacterial cells (diluted to 1:1,000) were inoculated into the center of the well using a sterile toothpick and incubated at 30 °C for 17 h. The mean diameter of the swim motility halo was measured using ImageJ (NIH) software.

### Pseudomonas cytotoxicity assay

A549 cells were seeded into 96-well flat-bottomed tissue culture plates a 1 × 10^4^ cells/well 24 hours prior to the start of the assay. PA strain PAK has been shown to express PcrV protein (GenBank: AAO91771.1) and was used in this assay to demonstrate anti-PcrV antibody-mediated protection from bacterial intoxication. PAK bacteria were grown overnight in Hy-Soy broth culture at 37°C in a shaking incubator. Bacteria from an overnight culture were pelleted by centrifugation, then suspended in PBS to an OD_600_ reading of 0.3. This sample was diluted 10-fold to give a bacterial suspension of 10^7^ CFU/mL. Pooled rabbit serum was titrated in serial twofold dilutions in PBS. Bacteria and serum were mixed 1:1 immediately before the start of the experiment. Growth medium was aspirated from the A549 cell monolayers, and 100 µL of bacterial sample was added to duplicate wells. The assay plate was incubated at 37°C and 5% CO_2_ for 90 min. The cell supernatant was aspirated from the wells, and 100 µL of fresh growth medium with the addition of 50 µg/mL gentamicin was added to the wells to kill remaining *Pseudomonas* and prevent further intoxication. Cells were incubated overnight at 37°C and 5% CO_2_ to allow toxin induced apoptosis to complete. The next day, 10 µL of a 1 mg/mL Resazurin solution was added to each well, and the plate was incubated for an additional 4 hours. Cells that remained viable metabolized Resazurin to produce a visible color change. This change was quantified by measuring the absorbance of the cell supernatant at 560 nm and 600 nm in a Molecular Devices Versamax microplate reader. The ratio of 560/600 nm absorbance is proportional to the number of viable cells remaining in each well of the assay.

### *Klebsiella* adherence inhibition

A549 cells were seeded as described for the cytotoxicity assay. Two *Klebsiella* strains were used in this assay, 4425 (O5, vaccine serotype) and 1015 (O4, non-vaccine serotype). Bacteria were grown on tryptic soy agar (TSA) plates overnight at 37 °C, then scrapped from the plate and suspended in PBS to an OD_600_ reading of 0.3. Pooled rabbit sera were diluted to varying concentrations in PBS, then mixed 1:1 with bacteria, and incubated at room temperature for 30 min. A549 cell monolayers were washed one time with PBS, and then 0.1 mL of sample was added. Cells were incubated at 37°C with shaking at 150 rpm for 90 min. After incubation, cells were washed eight times with 0.1 mL of PBS to remove non-adherent bacteria. Cells were treated with 0.1% Triton X-100 in water to solubilize the cell membrane and release adherent bacteria. Samples from the supernatant were plated on TSA plates, incubated overnight, and CFUs were enumerated the next day.

### Statistical analysis

Analyses for statistical significance were performed with GraphPad Prism v6.0 (GraphPad Software Inc., CA, USA). Survival curves were assessed by log-rank test. Comparisons for differences in functional antibody assays (motility inhibition, cell adherence, and cell cytotoxicity) were assessed by a two-tailed Student’s *t*-test. Statistical significance for ELISA titers were assessed by a two-tailed Mann-Whitney rank-sum test. *P*-values > 0.05 were considered as significant.

## Data Availability

The data that support the findings of this study are available from the corresponding author upon reasonable request.

## SUPPLEMENTAL MATERIAL

Supplemental material mbio.00807-25-s0001.docx Supplemental text, tables, and figures.

ASM does not own the copyrights to Supplemental Material that may be linked to, or accessed through, an article. The authors have granted ASM a non-exclusive, world-wide license to publish the Supplemental Material files. Please contact the corresponding author directly for reuse.
