# Supplementary material for: Novel Klebsiella pneumoniae and Pseudomonas aeruginosa MAPS vaccine combining O polysaccharides and pathogen-specific proteins
Source: mBio. 2025 Jun 26;16(8):e00807-25. doi: 10.1128/mbio.00807-25 (PMC12345170; doi:10.1128/mbio.00807-25)
Supplement: Supplemental material — Supplemental text, tables, and figures. [file mbio.00807-25-s0001.docx]

Supplementary Materials for

**Novel Klebsiella pneumoniae and Pseudomonas aeruginosa vaccine containing O polysaccharides and pathogen-specific proteins complexed with the Multiple Antigen Presenting System**

Mohammed N. Amin *et al.*

Corresponding author: Alan Cross, across@som.umaryland.edu

**The file includes:**

Figs. S1 to S3

Tables S1 and S2

**Fermentation of *Klebsiella* and *Pseudomonas* reagents strains and polysaccharide harvest.**

**Purification and characterization of bacterial polysaccharides.**

**Generation of protein expression plasmids for recombinant Rhizavidin fusion proteins.**

**Protein production and purification**

**Synthesis and characterization of scaffold polysaccharide constructs and MAPS complex formation.**

**KP-OPS or PA-COPS labeling with adipic acid dihydrazide (ADH):**

**Biotinylation of K19 CPS**

**Activation of Biotinylated K19**

**Scaffold polymer assembly**

**Analytical testing of scaffold**

**MAPS assembly and purification**

**References**

**Novel *Klebsiella pneumoniae* and *Pseudomonas aeruginosa* vaccine containing O polysaccharides and pathogen-specific proteins complexed with the Multiple Antigen Presenting System**

**Supplementary Materials**

Detailed description of the multivalent Klebsiella pneumoniae/Pseudomonas aeruginosa (KPPA) MAPS vaccine

Bacterial strains:

| **Table S1.** Bacterial strains used in this study | | |  |
| --- | --- | --- | --- |
| **Species** | **Strain** | **Source/characteristics (use)** | **Reference** |
| *K. pneumoniae* | CVD 3001 | O1:K- (reagent strain) | (1) |
| *K. pneumoniae* | CVD 3002 | O5:K- (reagent strain) | (1) |
| *K. pneumoniae* | 7380 | O2:K- (reagent strain) | (1) |
| *K. pneumoniae* | 390 | O3:K11 (reagent strain) | (1) |
| *K. oxytoca* | 160011 | O:K19 (reagent strain) | This study, Dr. J.  Kristie Johnson,  University of  Maryland Medical  Center |
| *K. pneumoniae* | B5055 | O1:K2 (challenge strain) | (2) |
| *K. pneumoniae* | TPEVGH-KPN-12 | O2:K2 (challenge strain), clinical isolate, Taiwan | This study Dr. YiTsung Lin, Taipei  Veterans General  Hospital and  National YangMing University,  Taiwan |
| *K. pneumoniae* | 700603 | KPO3 | ATCC |
| *K. pneumoniae* | 1015 | KPO4 | Statens Serum  Institut |
| *K. pneumoniae* | 4425 | KPO5 | (3) |
| *P. aeruginosa* | IATS O1 | O1:FlaA2 (reagent strain) | Dr. Joseph Lam,  University of  Guelph |

| *P. aeruginosa* | IATS O2 | O2: FlaB (reagent strain) | Dr. Joseph Lam,  University of  Guelph |
| --- | --- | --- | --- |
| *P. aeruginosa* | IATS O3 | O3:FlaB (reagent strain) | Dr. Joseph Lam,  University of  Guelph |
| *P. aeruginosa* | IATS O4 | O4:FlaB (reagent strain) | Dr. Joseph Lam,  University of  Guelph |
| *P. aeruginosa* | IATS O5 | O5:FlaB (reagent strain) | Dr. Joseph Lam,  University of  Guelph |
| *P. aeruginosa* | IATS O6 | O6:FlaA1 (reagent strain) | Dr. Joseph Lam,  University of  Guelph |
| *P. aeruginosa* | IATS O10 | O10:FlaB (reagent strain) | Dr. Joseph Lam,  University of  Guelph |
| *P. aeruginosa* | IATS O11 | O11:FlaB (reagent strain) | Dr. Joseph Lam,  University of  Guelph |
| *P. aeruginosa* | PAO1 | O5:FlaB (motility inhibition) | (1) |
| *P. aeruginosa* | 15-01737 | O3:FlaB (motility inhibition) | This study, clinical isolate  from UMMS provided by J. Johnson |
| *P. aeruginosa* | 14-04457 | O2/16:FlaB (motility inhibition) | This study, clinical isolate  from UMMS provided by J. Johnson |
| *P. aeruginosa* | PAK | O6:FlaA1 (cytotoxicity assays) | (1) |
| *P. aeruginosa* | M2 | O5:FlaB (challenge strain) | (1) |
| *P. aeruginosa* | SBI-N | O6:Fla? | Holder |
| *P. aeruginosa* | 1071 | PAO11 | Holder |
| *P. aeruginosa* | 15AP500428 | O4:Fla? | Karolinska  Hospital |
| *E. coli* | Origami B (DE3) *E. coli* (EMD Millipore) | Rhizavidin-FlaBD2-MrkA production strain | This study |
| *E. coli* | Origami B (DE3) *E. coli* (EMD Millipore) | Rhizavidin-FlaBD2-PcrV production strain | This study |

**Fermentation of *Klebsiella* and *Pseudomonas* reagents strains and polysaccharide harvest.** Strains were grown at 8L fermentation scale in a Bioflo 415 (Eppendorf, Germany). For this, bacteria were streaked from glycerol stocks to Hy-Soy agar plates and grown overnight at 37°C. Five to 10 individual colonies were then used to inoculate a 25 mL complete CDM culture in a 250 mL flask that was grown for 11-13 hours at 37°C/250 rpm. From this culture, 1 mL was then used to inoculate a 250 mL culture shake in a 2L flask in the same media that was then grown for 11-13 hours at 37°C/250 rpm. This was then used to inoculate the fermentation culture to an OD600 nm of 0.15. The fermentation culture was then grown for 18-24 hours to stationary phase at 37°C and 30% dissolved oxygen with 4LPM ambient air and correction to pH 7 with 28% ammonium hydroxide. Cultures were batch fed during mid-log stage with glycerol, trace elements and trace vitamins. At the end of the fermentation, cultures were processed for polysaccharide extraction as follows. For KP OPS extraction from LPS was accomplished by nitrous acid deamination as described, after transfer to glass bottles and incubation with 5-10 % acetic acid / 0.5% sodium nitrite pH 3.7 at 4°C under agitation for 16-24 hours. Extraction by this approach liberates the OPS without the core polysaccharide (4). Insoluble material and bulk biomass was then removed by centrifugation for 30 m at 10,000 *x g* at 4°C with a GS3 Rotor in a Sorvall RC5B. Post extraction supernatants (PES) were then brought to 1M NaCl and 0.2 um filtered by hollow fiber tangential flow filtration microfiltration (GE, NJ) passing the entire PES volume followed by an equivalent volume 1M NaCl flush. PA COPS was extracted as decribed^1^ by bringing the fermentation culture in the bioreactor to pH 3.7 with glacial acetic acid and incubation for 4 h at 100°C. Extraction by this approach releases the core and OPS (COPS) through hydrolysis of the labile bond between KDO and lipid A. Post hydrolysis supernatants (PHS) were processed by continuous centrifugation at 10k x G with a CEPA GLE (Eppendorf, Germany), and clarified by the same method used to filter KP PES. *K. oxytoca* K19 was obtained from the culture supernatant after bringing the complete culture to 20 mM EDTA, followed by centrifugation for 30 m at 10,000 *x g* at 4°C with a GS3 Rotor in a Sorvall RC5B. Cleared supernatants were further clarified by 0.45 μm filtration through a Polycap 150 TC filter.

**Purification and characterization of bacterial polysaccharides.** KP OPS was purified as described (1,4) by sequential TFF, anion-exchange and ammonium sulfate precipitation. Neutral PA COPS types (PA O4, O11) were purified by a similar approach as was detailed previously (1). Acidic PA COPS types (PA O1, O2, O3, O5, O6, O10) were purified as described below. Briefly, clarified PHS in 1 M NaCl was concentrated ~10-20-fold on either a 0.1 m^2^ 5 kDa Ultracell (Millipore, MA) or a 30 kDa Hydrosart (Sartorius, Germany) flat sheet TFF membranes at 14 psi TMP and diafiltered first 35-fold with 1 M NaCl followed by 10-fold diafiltration with 50 mM NaCl / 20 mM Tris pH 7. The TFF retentate was then bound to a Sartobind Q capsule (75 mL) at 45 mL/min with collection of the flow through and a subsequent flush with 5 bed volumes (BV) of 50 mM NaCl / 20 mM Tris pH 7. Bound COPS was eluted with 300 mM NaCl / 20 mM Tris pH 7. The 300 mM NaCl eluate fraction was then brought to 1.5- 1.9 M ammonium sulfate and incubated 16-24 hours at 4°C, followed by centrifugation at 10,000 *x g* at 4^o^C with a GS3 Rotor in a Sorvall RC5. The cleared supernatant was then filtered through 0.45 μm followed by concentration to 5-10 mg/mL by TFF on 2 x 200 cm^2^ 5 kDa Hydrosart membranes in a Slice 200 holder at 14 psi TMP followed by 10-fold diafiltration with DI water.

KO K19 CPS was purified from a 8L inactivated clarified K19 harvest by a series of sequential precipitations. The first step was a 3% CTAB precipitation of the CPS, the precipitate was redissolved in 1M CaCl2. This was followed with a 25% ethanol precipitation step to remove nucleic acids and proteins. An 80% ethanol step yielded a fairly pure CPS but still containing endotoxins activity. The CPS pellet from the 80% ethanol precipitation step was then delipidated and detoxified using 0.1 M NaOH in 95% ethanol at 37 C for 6.5hrs. The detoxified CPS solution was neutralized to pH 7 with 1 M acetic acid and the CPS was sedimented by centrifugation at 2,500 x g for 30 min at 4 ⁰C. The pellet was resuspended to 5-10 mg/mL based on pellet weight in 20 mM Tris, 1 M NaCl, pH 7.0 and stored at 4 ⁰C for ~72 hrs before being applied to a Sartobind Q membrane (Sartorius Stedim Biotech) and eluted from the positively charged membrane with 20 mM Tris, pH 7.0, 300 mMNaCl. The CPS solution was then diafiltered against 100 mM MES, 150 mM NaCl, pH 4.8 to concentrate and buffer exchange for final storage and use.

Residual impurities in purified polysaccharides were assessed for proteins by the bicinchoninic acid (BCA) assay (Thermo-Pierce, MA) per the manufacturer’s instructions using purified bovine serum albumin (Sigma Aldrich, MO) as standards. Residual endotoxin levels were measured using the LAL assay, and removal of nucleic acid was confirmed by absorbance at 260 nm.

**Generation of protein expression plasmids for recombinant Rhizavidin fusion proteins.**

An expression plasmid was designed and engineered to direct *E. coli* expression of a triple protein fusion designated Rhavi-FlaBD2-MrkA-his (Figure S1). The sequence of rhizavidin protein is highlighted in yellow, the sequence of the FlaBD2 protein is highlighted in pink and the sequence of the MrkA protein is highlighted in blue. The nucleotide sequence for *Rhizobium etli* rhizavidin (amino acids 45-179 lacking the signal sequence) was synthesized with an initiator methionine and codon optimized for *E. coli* expression and inserted into pET-21b plasmid under the control of the T7 polymerase promoter. Sequences to encode a 4 glycine and 3 serine linker (GGGGSSS) was inserted downstream and in-frame with the sequence to encode rhizavidin and followed by a sequence synthesized to express the domain 2 (D2, amino acids 170 – 386) of flagellin B (FlaB) from *Pseudomonas aeruginosa* PAO1 (Accession no. NP_249783). Nucleotides to encode an in-frame 4 alanine linker (AAAA) were inserted after FlaBD2 and then followed by sequence to encode a modified variant of *Klebsiella pneumoniae* MrkA with engineered donor strand complementation to improve stability of the monomer. The consensus amino acid sequence for MrkA (Accession no. AEO27492) was modified to remove amino acids

1 – 24, which encode a secretion signal. In addition, amino acids 25 – 44 were duplicated at the C-terminus of the sequence to enable donor strand complementation. A linker of 6 glycine residues was added just upstream of the donor complementation strand. The nucleotide sequence for this modified version of MrkA was synthesized codon optimized for *E. coli* expression and was followed with nucleotides to encode a GSG linker, a six histidine tag for purification, and a stop codon (Figure S2).

**Figure S1:**Schematic of Nosocomial KP/PA fusion proteins


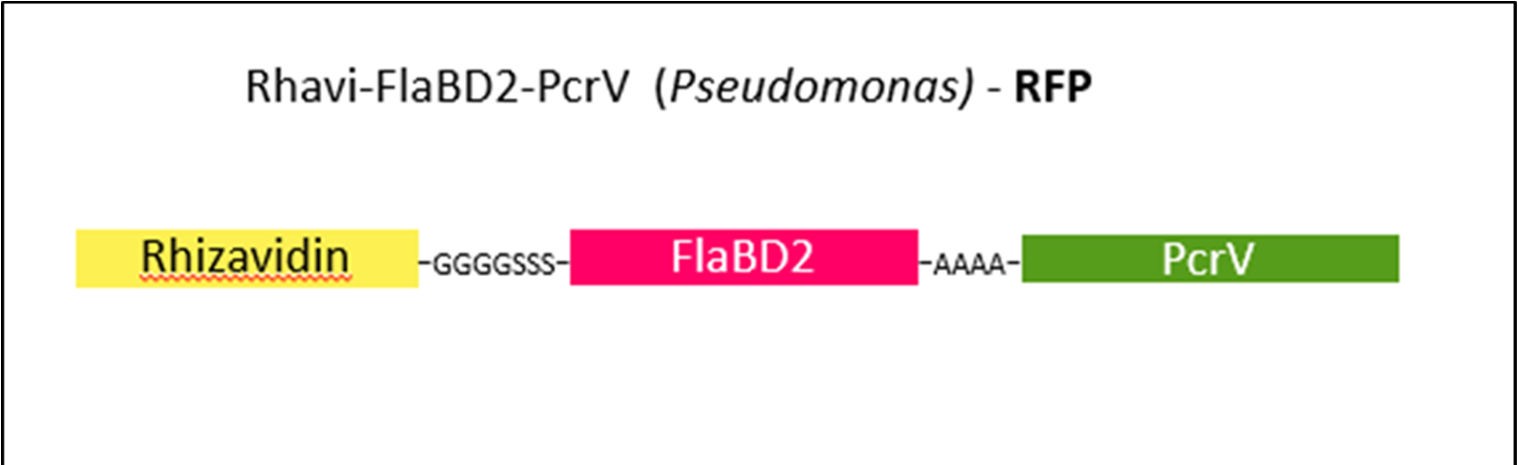

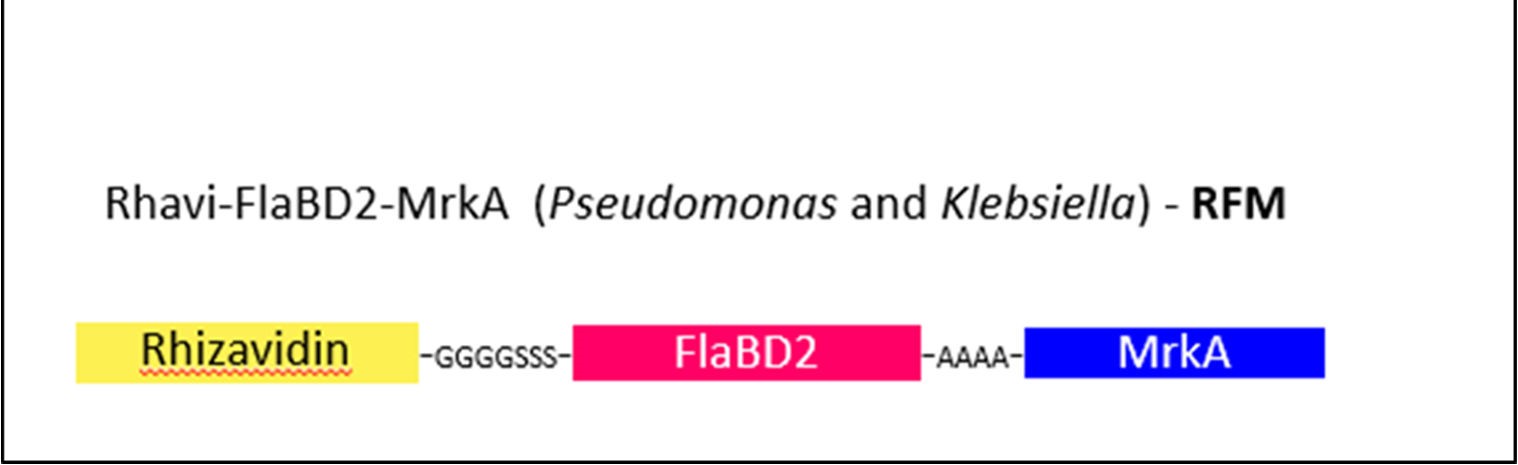


**Figure S2:** Amino acid sequence of Rhavi-FlaBD2-MrkA


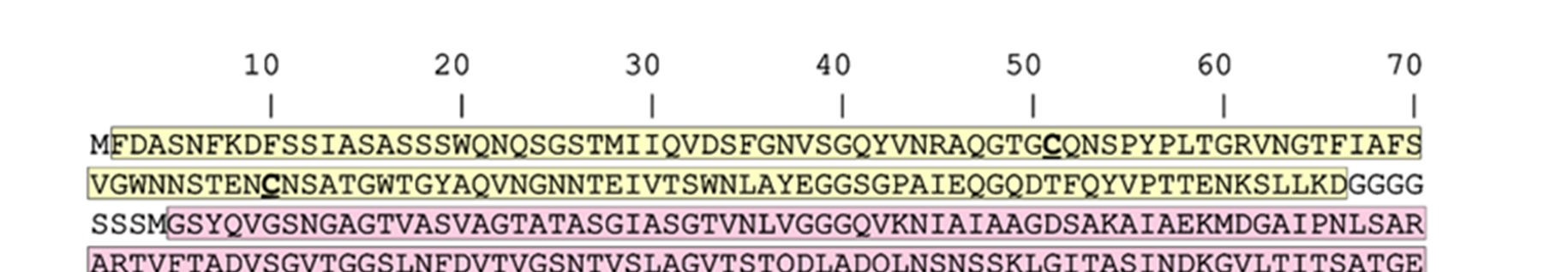

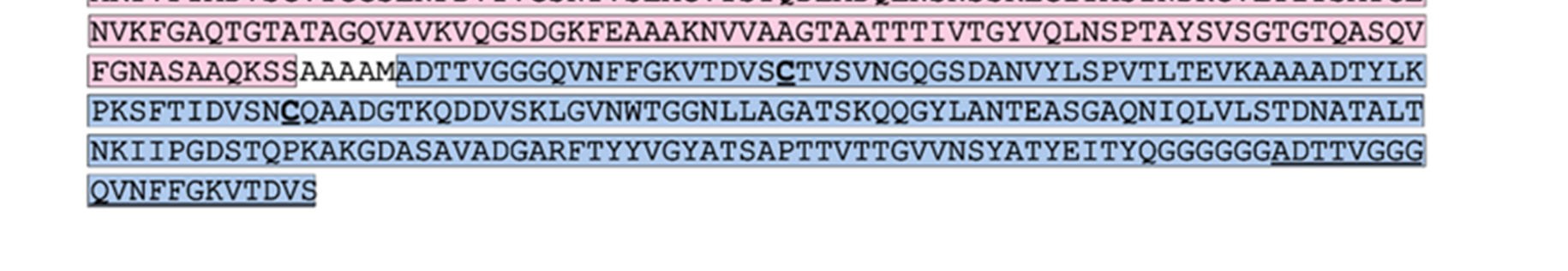


An expression plasmid was designed and engineered to direct *E. coli* expression of a triple protein fusion designated Rhavi-FlaBD2-PcrV-his (Figure S1). The sequences encoding rhizavidin, GGGGSSS linker, FlaBD2, AAAA linker were inserted into pET-21b as noted above. The *Pseudomonas aeruginosa* PcrV gene sequence was codon-optimized for *E. coli* based on a conserved amino acid sequence (Accession no. NP_250397) and inserted in-frame with the alanine linker and followed by the same GSG linker and histidine tag noted below. (Figure S3).

**Figure S3:** Amino acid sequence of Rhavi-FlaBD2-PcrV


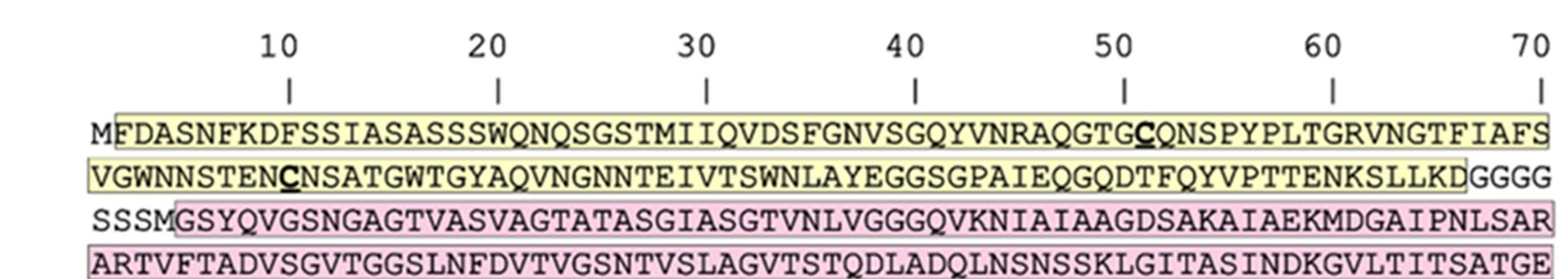

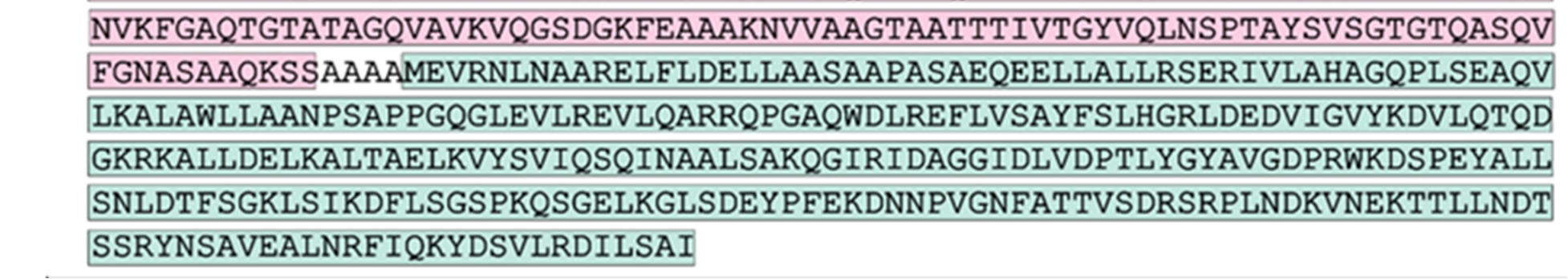


**Protein production and purification**

The plasmids encoding the rhizavidin fusion proteins were transformed into Origami B(DE3) *E. coli* (EMD Millipore) following the manufacturer’s protocol. A culture was initiated from a single colony and inoculated into Luria-Bertani (LB) medium containing carbenicillin (Carb+) for an overnight culture at 37° C. The next day, the starting culture was inoculated into a larger volume of LB/Carb+ and grown at 37° C until OD_600_ ~1.6 was reached. After cooling the culture to 16° C, IPTG was added to a final concentration of 0.1 to 1.0 mM. The induced culture was incubated at 16° C with shaking for 16 to 20 h. Bacteria were collected by centrifugation at 5000 x g for 20 min and the pellet was frozen at -20° C. To initiate purification, bacterial pellets were resuspended in 4 ml of chilled 20 mM Tris pH 8.0, 500 mM NaCl, 20 mM imidazole, 10 mM MgCl_2,_ and 2X Halt Protease Inhibitor (Thermo Fisher) per gram of cell pellet. The bacteria were disrupted with sonication or homogenization (Avestin Emulsiflex C5) followed by the addition of DNase I (Roche) to a final concentration of 25 µg/ml. Insoluble debris was removed by centrifugation at 5000 x g for 20 min. Cleared lysate was diluted with an equivalent volume of 20 mM Tris pH 8.0 with 500 mM NaCl to bring the final buffer concentration to 20 mM Tris, 500 mM NaCl, 10 mM imidazole, 5 mM MgCl_2,_ and 1x Halt Protease Inhibitor (Thermo Fisher). Proteins were purified by binding to nickel affinity resin (HisTrap HP prepacked column, Cytiva) at 4 degrees C, washing with 20 mM Tris, 500 mM NaCl, 20 mM imidazole and eluting bound protein with 20 mM Tris, 500 mM NaCl, 500 mM imidazole. The elution fractions with the target protein were further purified by size exclusion chromatography (Superdex 200 hiload 26/600, Cytiva) at 4 degrees C with 20 mM Tris, 150 mM NaCl, pH 8.0. The proteins were analyzed by SDSPAGE stained with simply blue (Thermo Fisher) and a BCA protein assay kit with the included protein standard (Pierce). Purified proteins were aliquoted, flash-frozen in liquid nitrogen, and stored at -80°C for future use.

**Synthesis and characterization of scaffold polysaccharide constructs and MAPS complex formation.**

**KP-OPS or PA-COPS labeling with adipic acid dihydrazide (ADH):** Purified PA COPS were first reacted with acetic anhydride to N-acetylate the amino groups present at the alanine and phosphoryl-ethanolamine moieties in the core polysaccharide. For this, individual COPS were brought to 10 mg/mL in 19mM sodium bicarbonate / 328 mM acetic anhydride pH 7 and incubated for 1 hour at RT at which point the sample was diluted 1:2.4 with 0.5M sodium bicarbonate and subjected to a 2^nd^ round of N-acetylation by addition of acetic anhydride to 135mM followed by incubation for 3 h at RT. Amino-blocked intermediates were then purified by TFF with 5 kDa Hydrosart 50 cm2 cassettes at 14 psi TMP, concentrating first to 10 mg/mL with subsequent 10-fold diafiltration against DI water followed by 10-fold diafiltration against 0.1M sodium acetate pH 5, and recovery after concentration to ~ 20 mg/mL. For activation of the KP OPS and amino-blocked PA core-OPS (COPS) for conjugation, they were derivatized at their reducing end 2,5-anhydro-D-mannose aldehyde and KDO ketone carbonyls respectively with adipic acid dihydrazide (ADH) that introduces a hydrazide amino group that can be used for subsequent linkage. For this, the COPS or OPS were brought to 10 mg/mL in 0.1 M sodium acetate / 20 mg/mL ADH pH 5 and incubated for 16-20 h at RT with stirring, at which point the reaction was then brought to 20 mg/mL sodium cyanoborohydride and incubated for 20-24 h at RT. Hydrazide labeled O-polysaccharides (OPS-AH) were then purified by first by 0.2um TFF microfiltration with 59cm2 mPES hollow fiber filters taking the permeate after 5-fold diafiltration against DI water, followed by TFF with 50 cm^2^ 5 kDa Hydrosart cassettes. The labeled OPS-AH were assayed for total carbohydrate by the Resorcinol assay, for free and bound amine groups by the TNBS assay, for size by SEC-HPLC using a triple PL-Aquagel-OH Columns: 60-50-40 linked in series; and for O-Acetylation levels (for PAO3, PAO6, PAO10 COPS) by the Hestrin assay.

**Biotinylation of K19 CPS:** K19 was first derivatized with biotin at K19 uronic acid carboxyls. Briefly, the purified K19 CPS was first concentrated to 10 mg/mL using a 5kDa Hydrosart TFF membrane (1 x 50 cm^2^ ) and then diafiltered 10-fold with 0.1 M MES /0.9% NaCl, pH 4.8. After being concentrated to 20 ± 5 mg/mL the retentate was collected and brought to a final concentration of 11 ± 1 mg/mL by diluting in 0.1 M MES/0.9% NaCl, pH 4.8. 1-Ethyl-3-(3dimethylaminopropyl) carbodiimide (EDC) (ThermoFisher) was then added to the K19 CPS solution at a ~2.25:1 EDC:CPS (wt/wt) ratio. After 5 minutes agitation with EDC, N-hydroxysuccinimide (NHS) (ThermoFisher) (5 mL of 50 mg/mL solution) was added under agitation at an approximate 0.5:1 NHS:CPS (wt/wt) ratio, and incubated 30 minutes at room temperature (RT). Biotin-PEG3-NH2 (Thermo) was then added in an approximate 0.24:1 Biotin:CPS (wt/wt) ratio, (5mL of 25 mg/mL biotin in DI water to 50 mL K19 NHS) and mixed under stirring. The pH was maintained between 7.1 – 7.3 by the addition of a 0.5 M NaHCO_3_ solution and the mixture incubated at RT for 18-24 hours. Unreacted chemicals and residual unconjugated biotin were removed after the reaction by TFF with a 5 kDa Hydrosart Membrane (1 x 50cm2) with 20-fold diafiltration against 0.1M Acetate buffer, pH 5.0 and concentration to 10 ± 5 mg/mL. The retentate was collected and the K19 CPS concentration determined by the Resorcinol assay using unmodified K19 CPS standards. The final purified biotinylated K19 (CPS-biotin) was analyzed for bound and free biotin, concentration, and molecular size distribution. Under this protocol a consistent level of ~ 0.03-0.06 mg biotin/mg K19 was achieved.

**Activation of Biotinylated K19:** Following biotinylation, CPS-biotin was subjected to a limited periodate oxidation. The 10 mg/L solution of CPS-Biotin was diluted to 5 mg/mL in 0.1 M NaOAc pH 5.0 buffer with gentle stirring. Sodium periodate was then added at a ratio of 0.15 mg per mg of CPS-Biotin. The reaction mixture was incubated with gentle stirring for 22h at RT in the dark, and the biotinylated-oxidized CPS (Bio-Ox-CPS) was then purified by TFF with a 50cm^2^ 5kDa Hydrosart Membrane against 0.1M NaOAc buffer, pH 5 to remove possible unreacted residual periodate. The retentate was further concentrated to 40 ± 5 mg/mL in the same buffer. After recovery of the retentate, the final material was brought to a 10% sucrose solution by diluting with a 40% sterile sucrose solution, yielding a concentration of Bio-Ox-CPS of 30 ± 5 mg/mL, and mixed for 5 min at RT. The Bio-Ox-CPS material was aliquoted and stored at -20 °C. The Bio-Ox-CPS purified material was analyzed by HPLC-SEC for molecular size using a triple SEC chromatography method using Agilent PL-Aquagel-OH columns: 60-50-40 linked in series, with PBS pH 7.4 buffer as the mobile phase at a flow rate of 0.5 mL/min and with monitoring using a refractive index RI410, and for UV absorbance at 280 nm and 252 nm. In addition, SEC-MALS was used to measure the absolute molecular weight of the K19 CPS before and after periodate oxidation of the biotinylated CPS (Bio-Ox-CPS). The aldehyde content was determined with an aldehyde assay (Sigma Colorimetric Aldehyde Assay Kit, Blue; Sigma-Aldrich Cat# MAK140) to confirm oxidation levels.

**Scaffold polymer assembly:** The concentrated Bio-Ox-CPS (30 mg/mL) was diluted to 25 mg/mL with 0.1 M NaOAc, pH 5.0 buffer in 10% sucrose. The concentrated Bio-Ox-CPS CPS and OPS/COPS-AH solutions, , were combined as follows: 1:1 Bio-Ox-CPS:COPS-AH (w/w) ratio for PAO1, O2, O3, O5, O6, O10 & O11 COPS; and 1:2 Bio-Ox-CPS:COPS-AH (w/w) ratio for PAO4 COPS-AH and all KP OPS-AH types. The combined solutions were mixed under gentle stirring overnight at RT. On day 2, using a 400 mg/mL NaBH_3_CN solution in water, 2 mg NaBH_3_CN per mg of Bio-Ox-CPS were added to the mixture and the reaction was carried out under stirring for 24 hrs at RT. On day 3 another 2 mg of NaBH_3_CN per mg Bio-Ox-CPS was added and the mixture was left to react another 24 hrs under stirring. On day 4, the reaction mixture was diluted to approximately 1-2 mg/ml total PS with 0.1M NaOAc, pH 5.0 and to cap the residual unreacted aldehydes, NaBH4 (25 mg/mL) in PBS buffer pH 7.4 was added to the reaction at 0.5mg NaBH_4_ per mg Bio-Ox-CPS under stirring for 2-3 hours at RT. The crude scaffold reaction mixture was stored at 4 ⁰C until final purification that was accomplished by dilution to 0.3 mg/mL and then 0.2 µm TFF microfiltration (110 cm^2^, Hollow Fiber Ultrafiltration Unit) with collection of the permeate. The microfiltration permeate was then concentrated to 2-4 mg/mL using a 50 kDa TFF membrane (155cm^2^, Hollow Fiber Ultrafiltration Unit) and diafiltered 20-fold against PBS, 10% sucrose pH 7.4. Finally the purified scaffold was concentrated to 6-10 mg/mL and aliquoted into sterile conical tubes and stored at -20 °C.

**Analytical testing of scaffold**: Molecular size distribution of the OPS K19 scaffold was determined by HPLC-SEC as described above using a PL-Aquagel-OH Columns: 60-50-40 linked in series.

A biotin assay (Pierce Biotin Quantitation Kit (Thermo Scientific Product) was used to determine the level of biotinylation in the final purified scaffold. In brief, HABA/Avidin premix is a reagent that enables an estimation of biotin to polysaccharide. HABA dye binds with Avidin to produce an orange color that can be absorbed at 500nm. Biotin displaces the HABA dye causing the absorbance to decrease. This change in absorbance is proportional to the amount of HABA displaced, and can be used to determine the amount of biotin in the polysaccharide sample. The OPS to K19 CPS output ratios in the purified scaffold was determined by 1H-NMR spectroscopy.(6)

1NMR sample preparation(6): Scaffold solutions in 10 percent sucrose containing ca. 5mg each total polysaccharide were first dialysed against DI water to remove sucrose and then freezedried. The freeze-dried samples were then exchanged 3 times against D2O 99.9% (Aldrich) using a SpeedVac vacuum concentrator (FisherThermo Scientific).

1H-NMR experiments (6): All 1H-NMR spectra were recorded on a Varian 400 MHz spectrometer at the CVD NMR facility in Baltimore, Maryland. The dried exchanged samples (1-5mg) were then dissolved in 99.99 D2O in 5-mm NMR tubes. 3-(Trimethylsilyl)-propionic-2,2,3,3-d4 acid sodium salt (TSP) (Aldrich) was then added as the internal reference for calibration of the spectrum (0 ppm). 1H-NMR spectra were recorded at 50⁰C for a total running time of 3 min (16 repetitions)

OPS to CPS output ratios calculation: Briefly, the molar ratio (mole:mole) of KP OPS to CPS in terms of repeating unit was determined using the peak area of specific proton reporter groups of the OPS repeat (anomeric protons with chemical shifts (δ) between 4.6-5.5ppm) versus reporter groups of the K19 CPS i.e. 6-deoxy methyl groups signals of the three rhamnosyl residues at around δ 1.3ppm. For the PA COPS K19 scaffold, specific PA COPS proton reporter groups such as the signals of N/O acetyl groups around δ 2.0 ppm were used for the calculations. The ratio of the measured number of OPS protons to the theoretical number of OPS protons yields the molar ratio of the OPS relative to 1 mole of K19 CPS (Table S2)

**Table S2:** COPS/OPS:CPS output w:w ratios in PA/KP Scaffolds as determined by 1H-NMR

| **Scaffold** | **COPS/OPS:CPS**  **w:w ratio** |
| --- | --- |
|  | **Lot #1 Lot #2** |
| **PAO1** | 0.71 0.99 |
| **PAO2** | 0.64 0.37 |
| **PAO3** | 1.20 0.93 |
| **PAO4** | 0.96 0.82 |
| **PAO5** | 1.43 1.08 |
| **PAO6** | 0.60 0.87 |
| **PAO10** | 0.72 0.59 |
| **PAO11** | 0.45 0.44 |
| **KPO1** | 0.97 0.71 |
| **KPO2** | 0.57 0.88 |
| **KPO3** | 1.20 1.05 |
| **KPO5** | 1.30 1.43 |

The levels of O-acetylation of the OPS in the scaffold (when applicable) was measured using

1H-NMR and/or the Hestrin colorimetric assay with acetylcholine chloride standards (Sigma Aldrich, MO). Total carbohydrate in the scaffold was determined by the resorcinol assay with purified individual OPS/CPS standards.

A competitive inhibition ELISA (CI-ELISA) was developed to assess OPS content and antigenicity of the scaffolds. Plates (96-well microtiter, Greiner Bio-one) were coated with 100 µL/well of the appropriate purified OPS at 0.25 µg/mL in PBS, pH 7.4 and were incubated for 16-20 h at 2-8 °C. The plates were washed 3 times with PBS + 0.05 % Tween 20 (PBS-T), and were then blocked with PBS, pH 7.4, with 5% Non-Fat Dry Milk for 1 h at 37 °C with shaking at 180 rpm on a standard microplate shaker. Test sample and purified OPS to generate a standard curve were diluted in 0.5% Non-Fat Dry Milk in PBS-T. The standard curve dilutions were chosen for each OPS to generate a linear standard curve and the test sample was diluted multiple times to ensure that a sample fell in the linear range of the standard curve. The blocked plates were washed 3 times with PBS-T and 50 µL/well of diluted sample and OPS standard curve was added to the plate with each in triplicate. The appropriate primary detecting antibody (Rabbit polyclonal anti-OPS) diluted 1/1000 in 0.5% Non-Fat Dry Milk in PBS-T was added at 50 µL/well diluted 1/95,000 in 0.5% Non-Fat Dry Milk in PBS-T and incubated for 1 h at 37 °C with shaking. The plate was washed 3 times with PBS-T followed by addition of 100 µL/well of the detecting reagent, TMB-ELISA substrate (Thermo Fisher) and 15 min incubation at room temperature in the dark. The reaction was quenched with 100 µL/well of 2N sulfuric acid and the absorbance at 450 nm was recorded using a Spectramax Microplate Reader (Molecular Devices). OPS concentration of test samples was calculated by interpolation of absorbance values of test samples into the log regression of the OPS standard curve utilizing Softmax Pro (Molecular Devices).

**MAPS assembly and purification**

MAPS complexes were generated by adding a rhizavidin fusion protein to the biotinylated polysaccharide scaffold in 20 mM Tris pH 8.0, 150 mM NaCl at the selected ratio (3:1 protein:PS w:w). For the KP MAPS complexes, fusion protein FlaBD2-MrkA was complexed with KPO1 and KPO3 OPS scaffolds and FlaBD2-PcrV was complexed with KPO2 and KPO5 OPS scaffolds. For the PA MAPS complexes, fusion protein FlaBD2-PcrV was complexed with PAO1, O2, O3 and O4 COPS scaffolds and FlaBD2-MrkA was complexed with PA O5, O6, O10 and O11 COPS scaffolds. The sample was incubated with end-over-end rotation at 25°C overnight followed by centrifugation at 10,000x g for 5 min to remove any insoluble material. The soluble material was collected and MAPS complexes were purified with size exclusion chromatography on a Superdex 200 column with 2 mM Tris, pH 8.0, 150 mM NaCl (5). Fractions were analyzed for protein content with SDS-PAGE of reduced samples with and without heating (98°C) and visualized with total protein Coomassie blue stain. The fractions containing MAPS complexes were identified from observation of the gel by the stained protein retention in large molecular weight complexes in the gel rather than the expected migration at the size of the protein dimer^4^ . The fractions containing the MAPS complexes were pooled and the protein/polysaccharide ratio of the MAPS was determined using the BCA protein assay kit (Pierce), and the resorcinol assay for total carbohydrate. The competitive inhibition ELISA assay (CI-ELISA) described above was used to determined the OPS content in the MAPS. The integrity of the MAPS complexes was evaluated with SDS-PAGE of reduced samples without heating visualized with total protein stain (5). The free protein content of the MAPS was quantitated by densitometry of the protein dimer content on the SDS-PAGE MAPS complex without heating compared to a standard amount of control protein dimer^4^ and determined to be less than 2% for all purified complexes. The molecular weight of the protein incorporated into the MAPS complexes was verified with SDS-PAGE of reduced, 98°C heated samples visualized with total protein stain and a molecular weight protein standard. All final purified MAPS were amenable to sterile-filtration with a 0.22 μm PES filter. Bacterial endotoxin levels (Charles River EndoSafe – PTS) was determined for each MAPS complex and the final total endotoxin level per rabbit immunization of the final formulated material was less than 55 EU per dose.

**References:**

1. Hegerle, N. *et al.* Development of a broad spectrum glycoconjugate vaccine to prevent wound and disseminated infections with *Klebsiella pneumoniae* and *Pseudomonas aeruginosa*. *PLoS One* **13**, e0203143 (2018).

2.Held TK, Mielke MEA, Chedid M, Unger M, Trautmann, Huhn D, Cross AS. G-CSF worsens the outcome of experimental Klebsiella pneumoniae through direct interaction with the bacteria. Blood 91:2525-35, 1998.

1. 3. Choi M, Hegerle N, Nkeze J, Sen S, Jamindar S, Nasrin S, Sen S, Permala-Booth J, Sinclair J, Tapia MD, Johnson JK, Mamadou S, Thaden JT, Fowler Jr VG, Aguilar A, Teran E, Decre D, Morel F, Krogfelt KA, Brauner A, Protonotariou E, Christaki E, Shindo Y, Lin Y-T, Kwa AL, Shakoor S, Singh-Moodley A, Perovic O, Jacobs J, Lunguya O, Simon R, Cross A, Tennant SM. The diversity of lipopolysaccharide (O) and capsular polysaccharide (K) antigens in invasive *Klebsiella pneumoniae* in a multi-country collection. *Front Microbiolo* 2020 Jun12:11:1249. Doi: 10.3389/fmicb.2020.01249.eCollection. PMID: 32595624

1. Vinogradov, E. et al. Structures of Lipopolysaccharides from *Klebsiella pneumoniae*. THE JOURNAL OF BIOLOGICAL CHEMISTRY **277**, 25070–25081 (2002).
2. Zhang, F. Lu, Y-J. and Malley, R. Multiple antigen-presenting system (MAPS) to induce comprehensive B- and T-cell immunity. PNAS **110,** (33)13564-13569 (2013).
3. Buerret M, Vignon M, Joseleau J-P. 1986. Structural investigation of the capsular polysaccharide from Klebsiella K19 by chemical and n.m.r. analyses. Carbohydr Res 157:13–25. https://doi.org/10.1016/0008-6215(86)85057-1
